# Synergistic Modulation of Microglial Polarization by Acteoside and Ferulic Acid via Dual Targeting of Nrf2 and RORγt to Alleviate Depression‐Associated Neuroinflammation

**DOI:** 10.1002/advs.202503889

**Published:** 2025-08-20

**Authors:** Dongjing Guo, Qiancheng Mao, Xinyu Fang, Liuxuan Huang, Haoquan Tian, Wenguang Yang, Feiyue Zhou, Ke Ma

**Affiliations:** ^1^ Shandong Co‐Innovation Center of Classic TCM Formula Shandong University of Traditional Chinese Medicine Jinan 250355 P. R. China

**Keywords:** acteoside/ferulic acid, depression, microglial phenotype, neuroinflammation, synergistic effect

## Abstract

Acteoside (ACT) and ferulic acid (FA), the principal bioactive constituents of Baihe Dihuang decoction (BDD), possess established anti‐inflammatory and antidepressant properties, but their combined effect on microglial phenotype modulation remains unclear. Integrated multi‐source data and machine learning identified ACT and FA as BDD's core components, mediating therapeutic effects via neurotransmitter regulation and inflammatory suppression. Co‐administering ACT and FA at their BDD ratio replicated the parent formulation’s anti‐inflammatory and antidepressant effects. Both compounds stabilized Nrf2, with ACT exhibiting greater potency. Crucially, the ACT/FA combination shifted microglia from pro‐inflammatory M1 to neuroprotective M2 phenotypes via dual activation of Nrf2 and RORγt pathways. Pharmacological inhibition or genetic knockdown of Nrf2 abolished these effects, confirming its central role. This dual mechanism concurrently rectifies neuroinflammation at its microglial source and impedes peripheral immune factor invasion, effectively restoring neuroimmune homeostasis in depression. These findings provide a mechanistic foundation for optimizing herbal‐derived combinatorial therapies targeting microglial polarization.

## Graphical abstract

Machine‐learning‐directed phytochemical pair ACT/FA, administered at BDD's orthodox ratio, synergistically modulates microglial polarization via dual Nrf2/RORγt targeting to attenuate neuroinflammation and depressive behaviors. This concentration‐dependent synergism replicates the parent formula's efficacy while providing a mechanistic basis for rational development of multi‐target phytomedicines against depression.

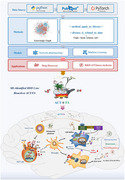

## 1 Introduction

Depression is a symptomatically heterogeneous disorder that encompasses emotional, cognitive, motivational, and physiological domains, and imposes a significant burden on individual health.^[^ ^1^ ^]^ The wide variability in clinical symptoms among affected individuals has, to date, hindered a precise understanding of the etiological factors and neural mechanisms driving its onset and progression. Although a range of different therapeutic approaches are available, their efficacy is often inconsistent, and treatment is often accompanied by adverse side effects, including nausea, insomnia, anorexia, headache, fatigue, and sexual dysfunction, as well as more severe complications such as intracerebral hemorrhage.^[^ ^2^ ^]^ Thus, there is an urgent need to unravel the pathological mechanisms underlying depression and discover novel and more effective antidepressant treatments.

Recent advances in immunological research have established a link between chronic psychosocial stress, microglial‐/macrophage‐induced inflammatory responses, and depression‐related behaviors.^[^ ^3^ ^]^ Under pathological conditions, microglial cells, the resident immune cells of the central nervous system (CNS), can rapidly undergo transition to an activated state, adopting either a “classic” pro‐inflammatory (M1‐like) phenotype or an “alternative activation” (M2‐like) phenotype.^[^ ^4^ ^]^ During development, chronic environmental stress and traumatic social experiences can contribute to a dysregulation of microglia, leading to neuroinflammation, impaired neurogenesis, synaptic abnormalities, and disruption of the excitation/inhibition (E/I) balance within brain regions, such as the medial prefrontal cortex (mPFC). Changes that can predispose individuals to the onset and progression of depressive disorders.^[^ ^5^ ^]^ Consequently, the regulation of microglial activation and function has emerged as a key system for developing novel treatments for depression.

Emerging evidence has enabled the identification of candidate genes and pathways involved in the modulation of microglial M1/M2 polarization that play pivotal roles in neuroinflammation.^[^ ^6^ ^]^ Among these, the IL‐17A cytokine produced by T helper 17 (Th17) cells has gained particular attention for its high pathogenic potential in depressive disorders. The chronic stress‐induced release of cytokines has been established to induce an upregulation of the Th17‐specific transcription factor retinoid‐related orphan receptor gamma t (RORγt), thereby promoting the differentiation of CD4+ T cells to Th17 cells in both the prefrontal cortex and peripheral tissues.^[^ ^7^ ^]^ Via a complex cascade of events, internal and external immune responses are propagated within the neural circuits of the emotional brain through a leaky blood‐brain barrier (BBB), thereby progressively exacerbating neuroinflammation and depressive symptoms.^[^ ^8^ ^]^ Notably, inhibiting RORγt has been shown to shift microglial polarization toward an anti‐inflammatory phenotype.^[^ ^9^ ^]^ Similarly, the transcription factor nuclear factor erythroid 2‐related factor 2 (Nrf2) has been found to modulate the M2 microglial phenotype by activating triggering receptors expressed on myeloid cell‐2 (TREM2), thereby alleviating depression‐like behaviors in animal models. Clinical and preclinical studies have consistently reported reduced Nrf2 expression in the mPFC of rodent models of depression and depressive patients.^[^ ^10^ ^]^ Mechanistically, Nrf2 directly regulates the transcription of TREM2 by binding to its promoter region.^[^ ^11^ ^]^ Collectively, the RORγt and Nrf2/TREM2 pathways have been established as key regulators of microglial activation and polarization.

In previous studies, we established that certain traditional Chinese medicine (TCM) formulations, notably Baihe Dihuang Decoction (BDD) and Baihe Zhimu Decoction (BZD),^[^ ^12^ ^]^ effectively mitigate immuno‐inflammatory responses in mood‐regulating neural circuits and intestinal tissues. These formulations were shown to alleviate neuronal damage, enhance synaptic plasticity, and normalize inflammatory cytokine/neuropeptide levels.^[^ ^13^, ^14^, ^15^ ^]^ Nevertheless, the phytochemical complexity of botanical medicines and fragmentary information regarding component–target interactions have to date hampered elucidation of bioactive constituents and their mechanisms of action. This fundamental limitation impedes the systematic assessment of synergistic antidepressant compounds in TCM. Consequently, resolving synergistic component combinations and delineating their cooperative mechanisms are imperative for developing mechanism‐driven, quality‐controlled TCM therapeutics with clinically validated efficacy.

An increasing number of ethnopharmacological studies have identified acteoside (ACT) and ferulic acid (FA) as the major bioactive compounds absorbed from BDD,^[^ ^16^, ^17^, ^18^ ^]^ and along with recent findings, our previous observations have tended to confirm that both ACT and FA have anti‐inflammatory and free radical‐scavenging properties, and have been shown to have therapeutic effects in depression models.^[^ ^19^, ^20^ ^]^ Notably, ACT or FA can mitigate neuronal injury in corticosterone (CORT)‐treated N2a cells and reverse chronic unpredictable mild stress (CUMS)‐induced depression‐like behavior in mice.^[^ ^21^, ^22^, ^23^ ^]^ However, it has yet to be established whether these compounds are more effective in combination in the treatment of depression and, if so, the neural mechanisms that underlie their potential synergistic effects warrant elucidation.

Evidence indicates that plant or herbal extracts generally have greater therapeutic efficacy than their isolated constituents at equivalent doses, which can probably be ascribed to synergistic interactions among the components.^[^ ^24^, ^25^ ^]^ It is thus conceivable that the synergistic activity of ACT and FA when co‐administered in their native proportions in BDD may replicate the antidepressant effects of the parent formulation. Guided by the principle of compatibility in TCM, we propose a research strategy for developing an active compound‐based Chinese medicinal formulation. Specifically, we hypothesize that a combination of ACT and FA, when administered at doses equivalent to their respective proportions in their source preparation, can alleviate prefrontal neuroinflammation and modulate microglial M1/M2 polarization by triggering Nrf2 and RORγt signaling pathways. Accordingly, in this study, we sought to determine whether the antidepressant efficacy of combinations of ACT and FA replicates that obtained using the parent formulation, and to elucidate the underlying mechanisms of their action on neuroinflammatory responses and microglial polarization.

## 2 Results

### 2.1 Combining Multi‐Source Data and Machine Learning Approaches to Predict Core Bioactive Components and Therapeutic Targets in BDD

**Figure 1:**
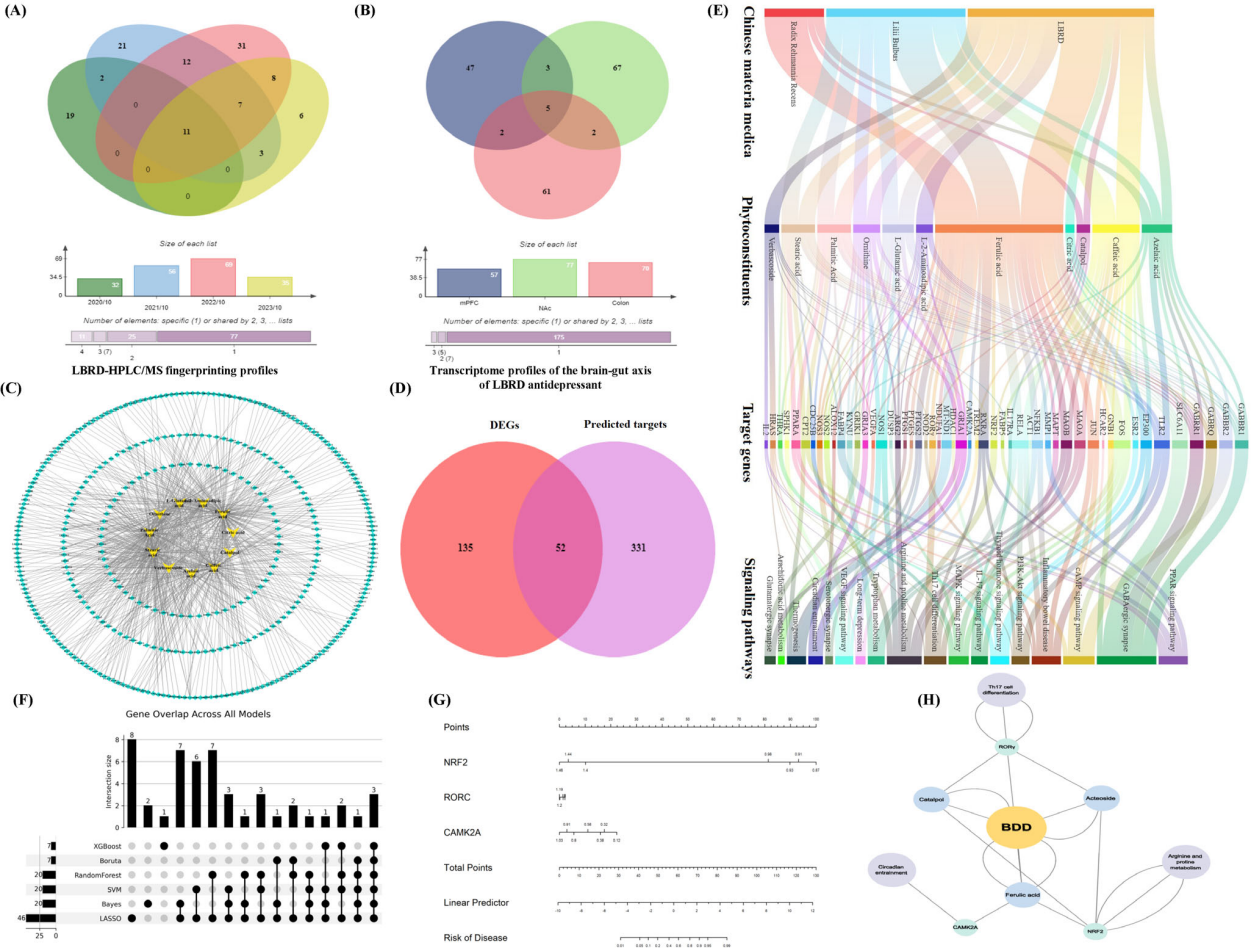
Combining multi‐source data and machine learning approaches to predict BDD's ACT/FA targeting immuno‐inflammatory pathways for depression. A) Venn diagram illustrating intersecting chemical ingredients across four Batches of Baihe Dihuang Tang (BDD) from 2020.10‐2023.10. B) Cross‐tissue transcriptomic convergence in the brain‐gut axis of BDD‐treated depression. C) Network pharmacology‐predicted putative targets of common bioactive components in BDD. D) A Venn diagram of intersecting targets emerged from BDD bioactive components versus RNA‐seq‐derived differentially expressed genes (DEGs) in BDD‐treated model. E) Integrated mechanistic network of BDD bioactive constituents‐antidepressant targets‐therapeutic pathways. F) Screening of key targets based on six machine learning algorithms. G) Nomogram for depression risk assessment and BDD therapeutic target validation. H) The summary of the association diagram of BDD most promising bioactive ingredients key targets for intervention in depression.

Previously, we characterized the chemical fingerprint profiles of four batches of Lily Bulb and Rehmannia decoctions (Table S1, Supporting Information) and accordingly identified the following 11 common components: azelaic acid, caffeic acid, catalpol, citric acid, FA, l‐2‐aminoadipic acid, l‐glutamic acid, ornithine, palmitic acid, stearic acid, and ACT (**Figure** **1**A). Using high‐throughput RNA sequencing, we profiled brain tissue transcriptomes from the mPFC, nucleus accumbens (NAc), and gut (colon) tissues (Table S2, Supporting Information) and identified 187 differentially expressed genes (DEGs) in the BDD‐treated depression model (Figure 1B). Among these, 52 core targets emerged from the intersection of common BDD‐depression targets and prefrontal cortex DEGs in BDD‐treated depressed mice (Figure 1C,D). Network analysis of the BDD‐active component‐target pathway network (Figure 1E) revealed significant enrichment in GABAergic synapses, Th17 cell differentiation, thermogenesis, the MAPK signaling pathway, and inflammatory cytokine pathways. To identify the key therapeutic targets associated with the core bioactive constituents of BDD, we employed a multi‐algorithm machine learning framework (Table S3, Supporting Information). On the basis of an integrative analysis using six machine learning models, we identified three candidate therapeutic targets, Nrf2, RORγt, and CAMK2A as potential mediators of the antidepressant effects of BDD (Figure 1F,G). In addition, by employing pharmacological classification and ADMET profiling, we identified ACT, FA, and catalpol as the top bioactive constituents associated with core therapeutic mechanisms of BDD. Subsequent validation based on neuronal network modeling provided confirmatory evidence to indicate ACT and FA as the most promising candidate constituents (Figure 1H). Collectively, we identified priority therapeutic targets through which ACT and FA, the core constituents of BDD, alleviate depression by regulating neurotransmitter synthesis and suppressing inflammatory responses.

### 2.2 The Antidepressant Effects of BDD are Mediated via Suppression of a Neurotoxic Microglia Phenotype in Response to Th17 Cell‐Driven IL‐17A/Act1/TRAF6 Signaling

**Figure 2:**
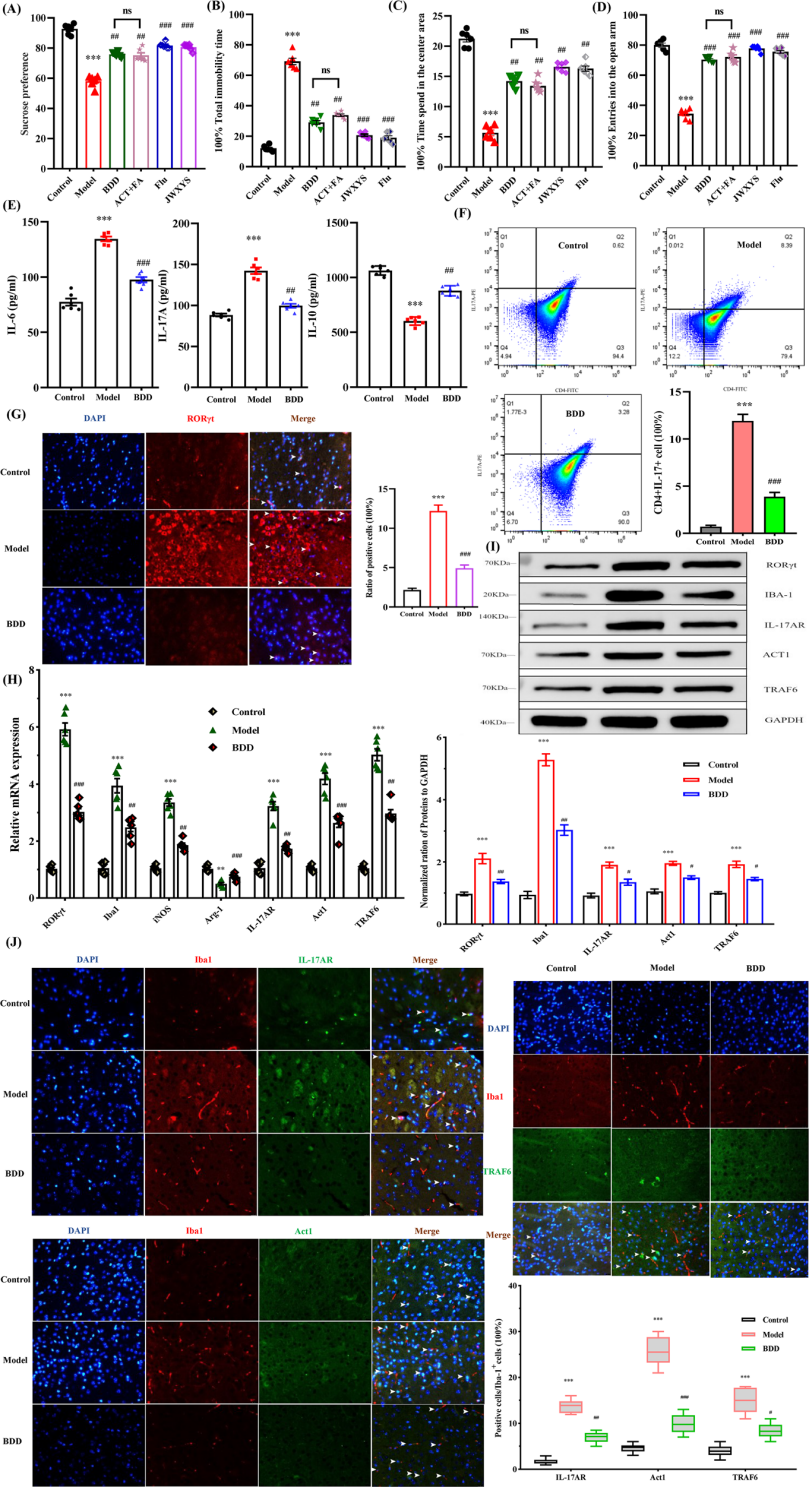
BDD exerts antidepressant effects by inhibiting the prefrontal Th17 cell‐driven RORγt /Act1/TRAF6 pathway mediates microglial activation. A) Sucrose preference (%) in the SPT. B) Immobility time in the FST. C) Retention time in the open field center. D) Percentage of retention time in the open arm. E) Levels of IL‐1β, IL‐17A, and IL‐10 in the mPFC of control or CUMS mice after treatment with saline or BDD. F) Representative flow cytometric analysis and statistical result of CD4+ Th17 cells contents within the mPFC. G) Fluorescence micrographs of DAPI (blue) and RORγt in the mPFC (×40 magnification, scale bar=10 µm). Quantitative analysis of RORγt, Iba1, iNOS, Arg‐1, IL‐17AR, Act1, and TRAF6 mRNAs H) level and protein expression of RORγt, Iba1, IL‐17AR, Act1, and TRAF6 I) in the mPFC of each group of mice. GAPDH was set as the internal control. Data for individual animals are displayed as mean ± SEM (n=6 per/group). J) Immunofluorescence double staining of retinal sections with Iba1 (blue), IL‐17AR (red), Act1 (red) or TRAF6 (red) and the ratio of IL‐17AR+ Iba1+ / Iba1+ cells, Act1+ Iba1+ / Iba1+ cells and TRAF6+ Iba1+ / Iba1+ cells. Three non‐overlapping fields of view were selected randomly in each slice under the fluorescence microscope. Arrows indicate the positive cells. Histogram is the quantification of the percentage of positive cells (n=3 per/group, 2 sections/animal). ^**^ *p*<0.01 and ^***^ *p*<0.001, compared with the control group; ^#^ *P*<0.05, ^##^ *P*< 0.01 and ^###^ *P*<0.001, compared with the CUMS‐induced depressive like behavior group.

On the basis of traditional Chinese medicine compatibility and potential medicinal uses, we established ACT and FA to be representative active compounds in the Chinese medicinal formulation BDD. We then sought to determine whether the antidepressant effects of BDD are associated with the regulation of microglial phenotypes. Mice in the BDD group were treated with three different concentrations of BDD dissolved in saline at 9.37, 6.25, and 4.16 g/kg/day administered intragastrically for three consecutive weeks (Figure S1, Supporting Information). We accordingly identified 6.25 g kg^−1^ as the optimal effective drug concentration for subsequent mechanistic analyses. Using different behavioral phenotypic parameters, we found that repeated administration of BDD had a pronounced effect on depressive or anxiety‐like behavior of mice exposed to CUMS (**Figure** **2**A–D). Interestingly, the antidepressant efficacies of ACT and FA were comparable to those obtained using BDD. Given provisional evidence linking microglial activation and changes in fronto‐limbic volume in depression models, we analyzed changes in the mPFC. ELISA of mPFC supernatants revealed elevated levels of the pro‐inflammatory factors IL‐6 and IL‐17A and reductions in the levels of IL‐10 in CUMS‐exposed mice. Notably, treatment with BDD was found to be associated with a significant downregulation in the expression of pro‐inflammatory factors and upregulated expression of anti‐inflammatory factors (Figure 2E).

The prolonged production of IL‐17A by Th17 cells has been established to be dependent on the transcription factor RORγt. We found that chronic BDD treatment reduced the population of CD4 + IL‐17 + Th17 cells in the mPFC, thereby reversing the CUMS‐induced elevation in the production of IL‐17A (Figure 2F). Immunofluorescence staining, qRT‐PCR, and western blot analysis confirmed that treatment with BDD contributed to a downregulation in the expression of RORγt (Figure 2G–I). To further examine the neuroprotective effects of BDD on IL‐17A‐mediated microglial activation, we performed qRT‐PCR, western blotting (the whole uncropped images of the original western blots in Figure S2, Supporting Information), and immunofluorescence staining. BDD treatment was found to reduce expression of the microglial activation markers Iba1 and iNOS, whilst promoting an increase in the expression of Arg‐1. Moreover, BDD was shown to suppress the CUMS‐induced upregulation of IL‐17AR, Act1, and TRAF6 (Figure 2H,I). Consistent with these findings, immunofluorescence double staining for Iba1 and IL‐17A signaling markers confirmed that treatment with BDD reduced the levels of IL‐17AR, Act1, and TRAF6 in mPFC microglia (Figure 2J). Collectively, these findings provide convincing evidence to indicate that BDD containing ACT and FA restores activated microglial homeostasis mediated via the Th17 cell‐driven RORγt/Act1/TRAF6 signaling pathway to ameliorate chronic stress‐induced depression.

### 2.3 Identification of Nrf2 as a Direct Binding Protein for ACT and FA

**Figure 3:**
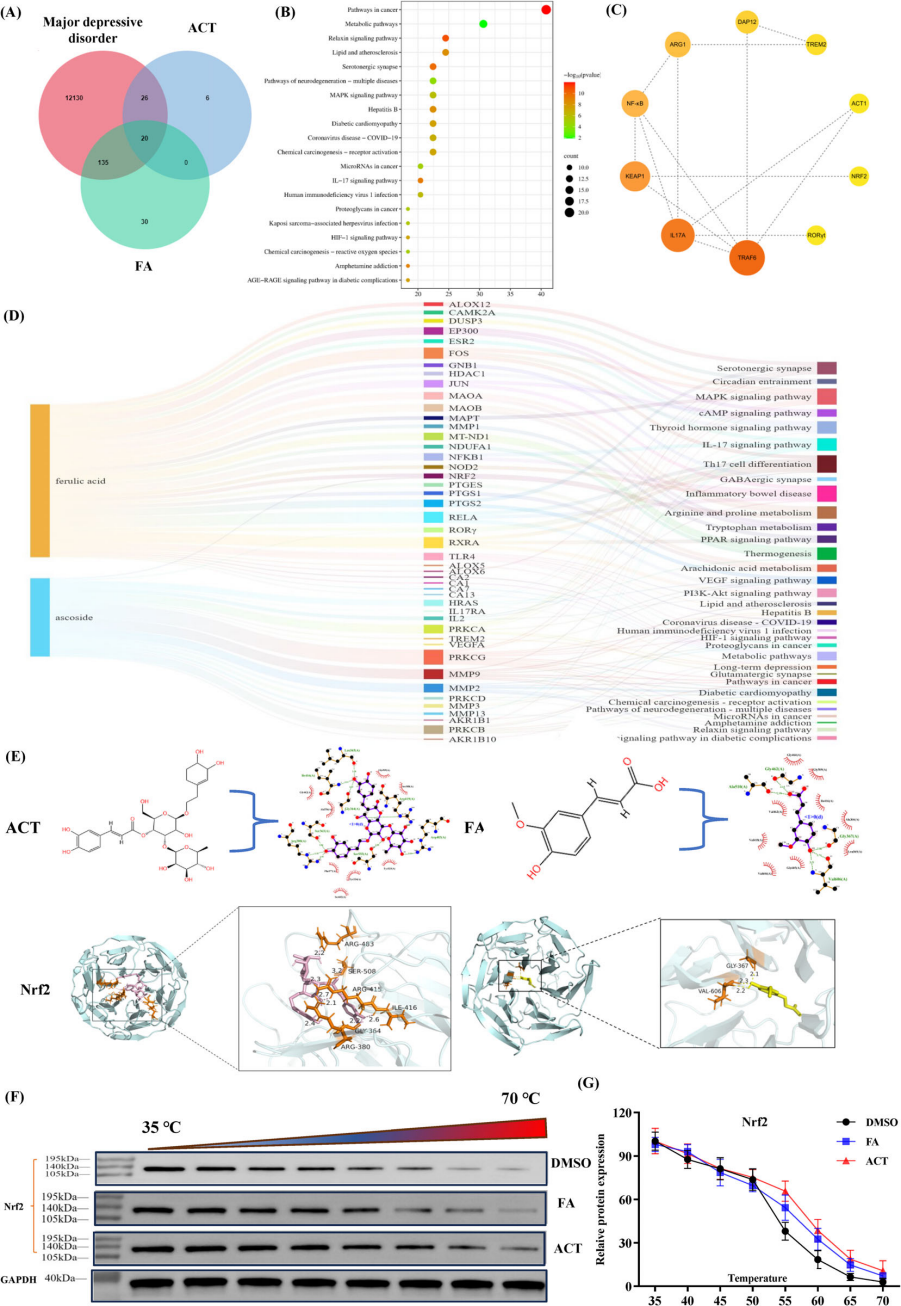
Identification of Nrf2 as a direct binding protein for ACT and FA. A) Venn diagram illustrating the overlap of small molecules and proteins associated with major depressive disorder and predicted binding targets of Acteoside (ACT) and Ferulic Acid (FA). B) Signaling pathways and targets of ACT and FA linked to antidepressant effects. C) Protein‐protein interaction (PPI) network showing correlations among common targets. D) Sankey diagram visualizing relationships between ingredients, targets, and pathways. E) Molecular docking of active components with core targets. Key residues are highlighted: ACT (light pink), FA (yellow), and binding partners (orange). Nrf2 is shown in pale cyan; RORγt in blue‐white. F) Nrf2 protein expression assessed by cellular thermal shift assay. G) Nrf2 protein expression detected by Western blot in BV2 cells. Data are presented as mean ± SEM (n=3).

To validate the direct interaction between the synergistic constituents of BDD (ACT and FA) and Nrf2, we adopted a combined network pharmacology and molecular docking approach, performed in conjunction with cellular thermal shift assay (CETSA) analyses. Active constituent–target network analysis revealed that ACT and FA target molecules involved in pathways considered essential for the treatment of depression, including metabolic pathways, MAPK signaling, IL‐17 signaling, neurotrophic signaling, neurotransmitter synthesis, cAMP signaling, and inflammatory cytokine pathways (**Figure** **3**A–D). Among the common targets of ACT and FA, we singled out Nrf2, TREM2, RORγt, and IL‐17AR, each of which is implicated in the regulation of microglial M1/M2 polarization, for the construction of a PPI network and molecular docking analysis.

The docking results indicated that ACT and FA bound strongly to these targets, with binding energies <‐5.0 kcal⋅mol^−1^. Specifically, we obtained binding energies of ≤ −6.6, ≤ −5.7, and ≤ −7.2 kcal/mol for interactions between the two core constituents and Nrf2, TREM2, and RORγt, respectively, indicating high stability. The 3D image results revealed that both ACT and FA molecules can insert into the docking pockets of Nrf2, TREM2, and RORγt. Interestingly, these molecular docking studies predicted substantially lower energies for the binding of ACT and FA to Nrf2 than to either TREM2 or RORγt (Figure S3, Supporting Information). Moreover, the more negative value obtained for ACT indicated its stronger binding to Nrf2, compared with FA (Table S4, Supporting Information). Structural analysis indicated that the hydroxyl groups and oxygen atoms of ACT serve as both donors and acceptors that facilitate the formation of hydrogen bonds with specific amino acid residues of Nrf2, including GLY‐364, ARG‐380, ARG‐415, ILE‐416, ARG‐483, and SER‐580. Furthermore, the aromatic ring of ACT facilitates hydrophobic interactions with Nrf2. Comparatively, FA was established to form hydrogen bonds with GLY‐367 and VAL‐606 residues of Nrf2 (Figure 3E). Enlarged 2D images revealed that both ACT and FA interact favorably with the DNA‐binding domain (residues 364 to 416) of Nrf2, thus indicating that simultaneous interactions between ACT and FA and Nrf2 contribute to enhancing the stability of this target protein.

To further confirm these interactions, we performed a cellular thermal shift assay (CETSA) to evaluate the binding affinities of ACT and FA for Nrf2. Both ACT and FA were demonstrated to have significant stabilizing effects on Nrf2, with ACT being found to have a stronger effect, as evidenced by a greater rightward shift in the Nrf2 melting curve compared with FA (Figure 3F,G). These findings thus provided evidence to indicate that ACT and FA may have neuroprotective and anti‐inflammatory effects by modulating the neuroinflammatory microenvironment, mediated via Th17 cell‐driven RORγt activation and the Nrf2/TREM2 signaling pathway.

### 2.4 The Combined Administration of ACT and FA Alleviates CUMS‐Induced Anxiety/Depression‐Like Behaviors

**Figure 4:**
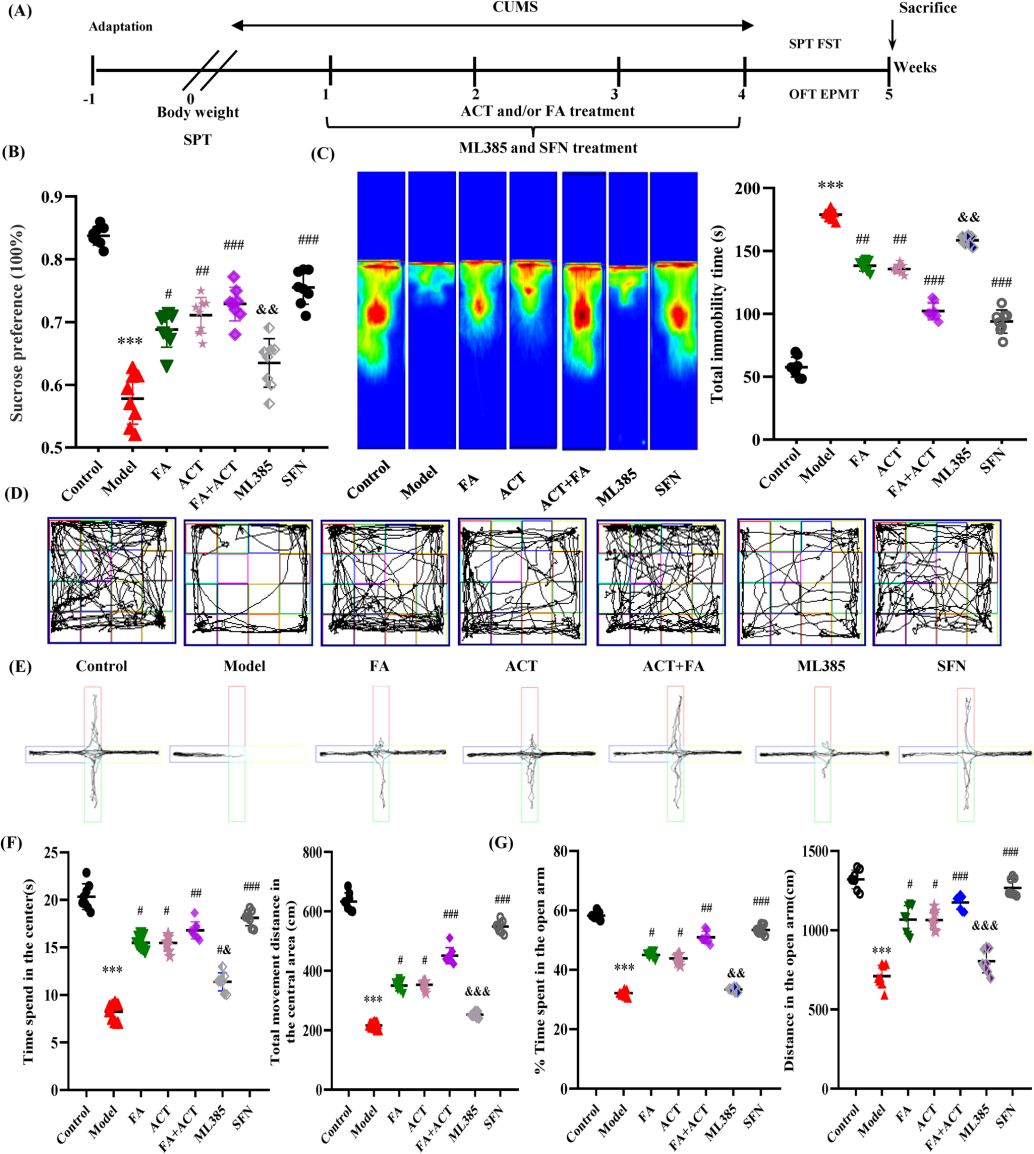
Synergistic antidepressant and anti‐inflammatory effects of ACT and FA. A) Experimental timeline for the study procedures. B) ACT and FA combination improved sucrose consumption in CUMS‐induced depressive behaviors in the sucrose preference test. C) Thermal imaging of mice trajectories and immobility time in the forced swim test. D) Representative movement tracks in the open field test. E) Trajectory diagrams of mice in the elevated plus maze test. F) Central zone residence time and total distance in the OFT. G) Retention time in the open arm and movement distance in the EPMT. Data are presented as the mean ± SEM (n=6–8 per/group). ^**^ *p*<0.01 and ^***^ *p*<0.001, compared with the control group; ^#^ *p*<0.05, ^##^ *p*<0.01 and ^###^ *p*<0.001, compared with the CUMS‐induced model group; ^&^ *P*<0.05, ^&&^ *P*<0.01 and ^&&&^ *P*< 0.001, versus the ACT+FA group.

To determine whether the predicted synergism between ACT and FA ameliorates depression‐like behaviors, we performed four classical behavioral assays using a mouse model of CUMS‐induced depression. Mice were initially exposed to CUMS for 4 weeks, followed by treatment for 3 weeks with an Nrf2 activator (SFN), an Nrf2 inhibitor (ML385), ACT, FA, or ACT + FA (**Figure** **4**A). Behavioral assays were initially conducted to evaluate the effects of single treatments with ACT or FA. Following CUMS induction, ACT at doses of 30 and 60 mg kg^−1^, although not 90 mg kg^−1^, were found to promote a significant enhancement in sucrose preference and reduced immobility time in the forced swimming test (Figure S4, Supporting Information). On the basis of these observations, we selected a concentration of 60 mg kg^−1^ as the optimal dose of ACT for subsequent experiments. Compared with fluoxetine, used as a control standard drug, FA treatment was found to promote a significant increase in sucrose consumption and reduced immobility time when administered at 40 mg kg^−1^. Subsequent phytochemical analysis revealed ACT and FA to be enriched in BDD, with a respective concentration ratio of 9 to 1. Further behavioral analyses, in which we compared the antidepressant responses to ACT and FA at different ratios (ACT:FA = 9:1, 5:5, and 1:9; 45 mg ACT+5 mg FA, 25 mg ACT+25 mg FA, and 5 mg ACT+ 45 mg FA) indicated that the antidepressant effects of ACT and FA at the equivalent dose ratio found in BDD for each compound (ACT:FA = 9:1, ACT 45 mg kg^−1^ combined with FA 5 mg kg^−1^) was superior to that of the other ratios (Figure S5, Supporting Information).

Finally, we validated the synergistic effects of ACT and FA against depression associated with the activation of Nrf2. As shown in Figure 4B–G, in response to treatment we detected significant changes in anhedonia, behavioral despair, and exploratory capacity in CUMS‐induced mice, reflected in a reduction in sucrose preference and retention time in the open field test, prolonged immobility time in the forced swimming test, and lower elevated plus maze duration of movement in the open arm. However, we found that the depression and anxiety‐like symptoms induced by CUMS were effectively ameliorated by administration of the chronic Nrf2 activator SFN, or ACT and/or FA treatment. Notably, the co‐treatment with ACT and FA at a ratio of 9:1 had stronger effects compared with either ACT or FA treatment administered alone (Figure 4B–G). Moreover, co‐administration of the Nrf2 inhibitor ML385 partly abrogated the benefits obtained in diminishing depressive‐ and anxiety‐like behaviors attributable to the compatibility between ACT and FA. These findings accordingly provide convincing evidence that ACT and FA synergistically exert antidepressant effects by promoting the activation of Nrf2, thereby providing insights for their potential therapeutic applications.

### 2.5 Synergism Between ACT and FA Induces the Switch of Prefrontal Microglia from a Pro‐Inflammatory to Anti‐Inflammatory Phenotype via an Nrf2‐Dependent Pathway

**Figure 5:**
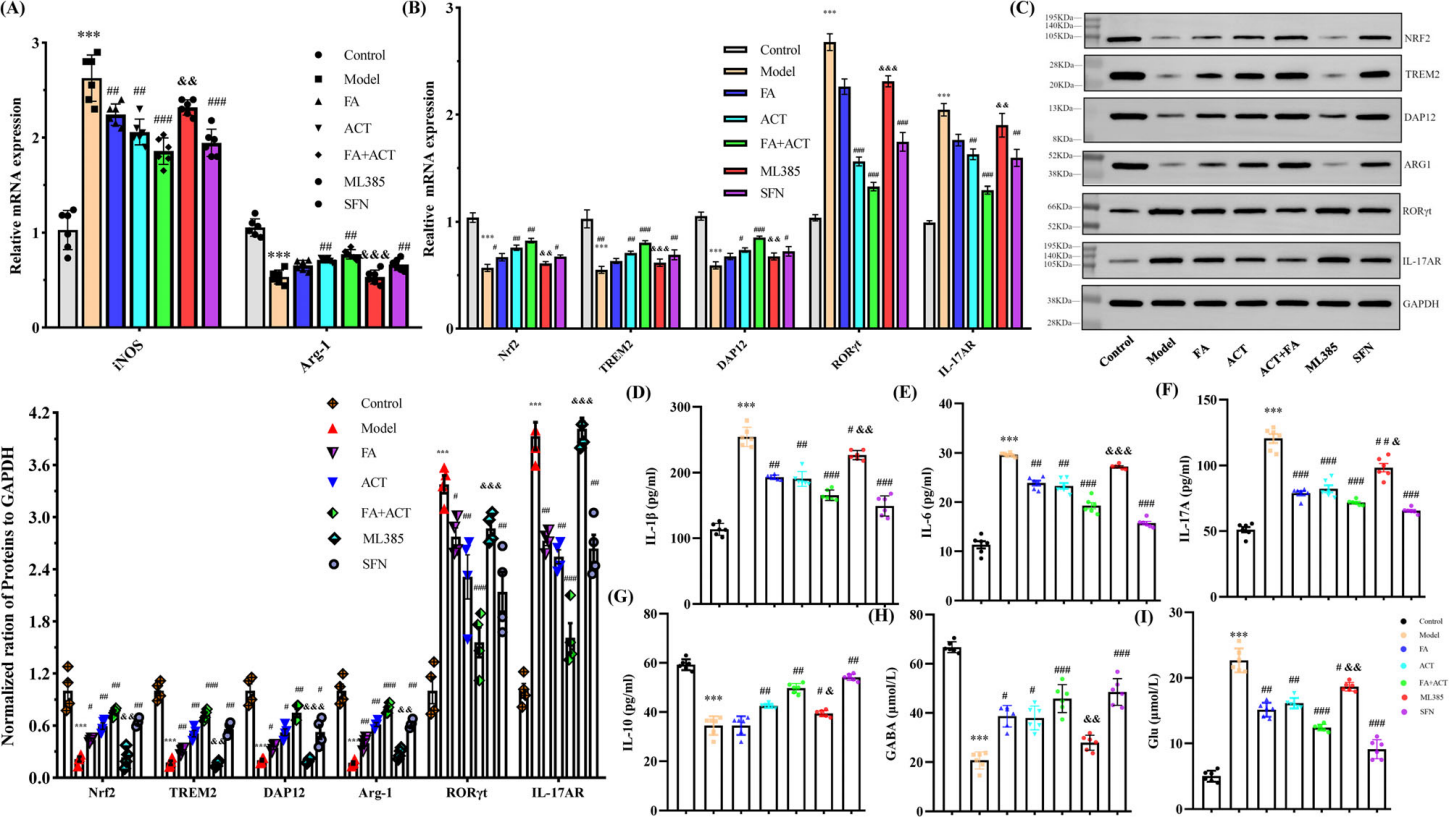
ACT combined with FA modulates microglial polarization via Nrf2 activation and RORγt/IL‐17AR Signaling. A) Expression of M1 (iNOS) and M2 (Arg‐1) microglial polarization markers in the prefrontal cortex. B) Quantitative analysis of Nrf2, TREM2, DAP12, RORγt, and IL‐17AR mRNA expression in the mPFC. C) Western blot of Nrf2/TREM2/DAP12/Arg‐1 and RORγt/IL‐17AR protein levels; GAPDH served as the internal control. D–I) Cytokine and neurotransmitter levels: IL‐1β (D), IL‐6 (E), IL‐17A (F), IL‐10 (G), GABA (H), and Glu (I). Data are presented as the mean ± SEM (n=6 per/group). ^**^ *p*<0.01 and ^***^ *p*<0.001, compared with the control group; ^#^ *p*<0.05, ^##^ *p*<0.01 and ^###^ *p*<0.001, compared with the CUMS‐induced model group; ^&^ *P*<0.05, ^&&^ *P*<0.01 and ^&&&^ *P*<0.001, versus the ACT+FA group.

Microglia adopt two primary activation states, namely, the classically activated M1 phenotype, characterized by pro‐inflammatory mediators, such as iNOS, and the alternatively activated M2 phenotype, characterized by anti‐inflammatory mediators, including arginase 1 (Arg‐1). Chronic stress typically contributes to pronounced increases in the expression of M1‐associated inflammatory mediators, including iNOS, whereas it tends to cause reductions in the expression of M2‐associated markers, such as Arg‐1, in the mPFC. We found that treatment with either ACT or FA, administered alone or in combination, reversed the CUMS‐induced upregulation of iNOS mRNA expression and enhanced Arg‐1 expression in the mPFC (**Figure** **5**A). Conversely, we demonstrated that the synergistic effects of ACT and FA can be reversed by blocking the Nrf2 pathway using the inhibitor ML385. We examined the effects of combined treatment on the microglial expression of M1 and M2 polarization markers, the results of which were consistent with those obtained at the mRNA level using qRT‐PCR.

Given the pivotal role played by Nrf2/TREM2 signaling in anti‐inflammatory microglial phenotypes, we investigated whether the synergistic effect of ACT and FA influences the switching of prefrontal microglial phenotypes via this pathway. It was accordingly found that exposure to CUMS was associated with significant reductions in the expression of Nrf2, TREM2, its adaptor protein DNAX activation protein 12 kDa (DAP12), and Arg‐1 in the mPFC, which were partially reversed by the combined treatment with ACT and FA (Figure 5B,C). However, inhibition of Nrf2 signaling using ML385 abolished the capacity of the ACT‐FA combination to upregulate TREM2/DAP12/Arg‐1 expression. Conversely, by upregulating the TREM2/DAP12/Arg‐1 pathway, the activation of Nrf2 by SFN was associated with increases in the levels of Arg‐1, thereby inducing an anti‐inflammatory microglial phenotype. Additionally, in response to exposure to CUMS, we detected a significant increase in the expression of RORγt and IL‐17AR mRNAs in the mPFC, a trend mirrored at the protein level (Figure 5C), as confirmed by western blot analysis (the pattern plot of the protein band was derived from the whole un‐cropped images of the original western blots in the Figure S6, Supporting Information). Notably, compared with ACT or FA administered alone, the combined treatment with ACT and FA produced a more pronounced downregulatory effect on RORγt and IL‐17AR. These findings thus tend to indicate that the neuroprotective effects of the ACT‐FA combination may involve modulation of the Nrf2 signaling pathway coupled with RORγt‐driven IL‐17A expression.

To further investigate the relationship between the anti‐inflammatory effects of the ACT‐FA combination and Nrf2‐mediated regulation of inflammatory factors, we measured the levels of IL‐1β, IL‐6, IL‐17A, IL‐10, and the neurotransmitters GABA and glutamic acid in mouse serum using ELISA. Compared with the control mice, exposure to CUMS was found to promote significant reductions in the levels of GABA and IL‐10, whilst inducing increases in the levels of IL‐1β, IL‐6, IL‐17A, and glutamic acid, whereas treatment with ACT and/or FA reversed these changes, indicating that these phytoconstituents can contribute to preventing a pro‐inflammatory switch and restored the E/I balance disrupted by CUMS. However, these effects were suppressed in response to the inhibition of Nrf2 signaling by ML385 (Figure 5D–I). Taken together, these findings indicate that FA and ACT maintain the balance of microglial M1/M2 polarization in a collaborative manner to attenuate prefrontal neuroimmune inflammatory responses in CUMS‐induced depression by suppressing RORγt/IL‐17AR signaling and promoting the Nrf2/TREM2/DAP12/Arg‐1 pathway.

### 2.6 Nrf2 is Necessary for the Neuroprotective and Anti‑Inflammatory Effects of the Combined Activity of ACT and FA

**Figure 6:**
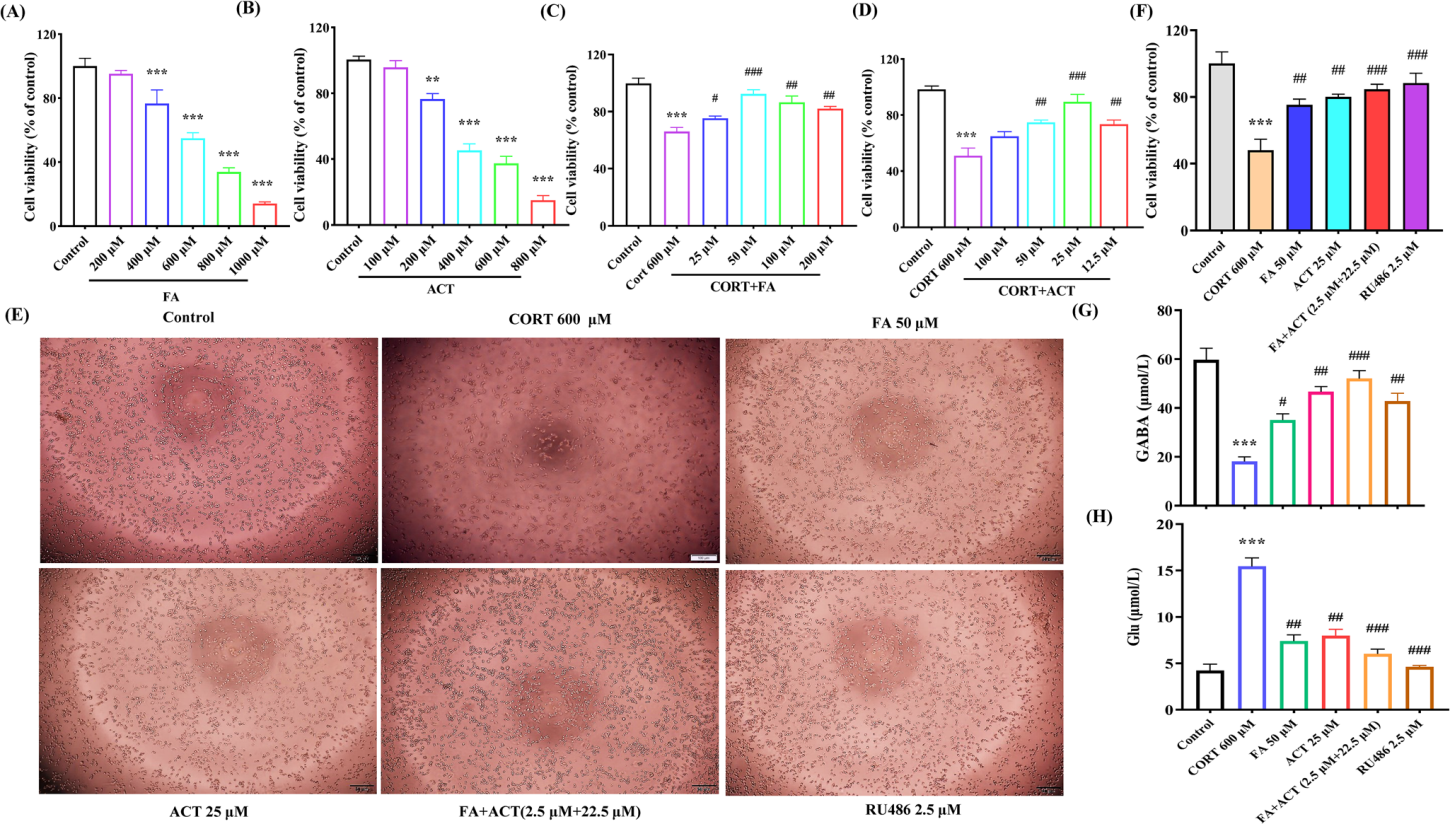
Protective effect of ACT and FA on CORT‐induced cytotoxicity. A) A,B) CCK‐8 assay results in Neuro‐2a cells treated with different concentrations of FA and ACT for 24 h. C,D) CCK‐8 assay results in Neuro‐2a cells treated with different concentrations of FA or ACT for 30 min, then 600 µm CORT for 24 h. E) Histological changes in nerve cells damaged by CORT and treated with ACT combined with FA. F) Statistical analysis of co‐treatment of ACT and FA with the equivalent ratio of them in the BDD against CORT‐induced toxicity. H,G) The expression of GABA and Glu in the supernatant of N2a cells. Results are presented as the mean ± SEM of three independent experiments. ^*^ *p*<0.05, ^**^ *p*<0.01 and ^***^ *p*<0.001, compared with the control group; ^#^ *p*<0.05, ^##^ *p*<0.01 and ^###^ *p*<0.001, compared with the CORT‐induced cell model group; ^&^ *P*<0.05, ^&&^ *P*<0.01 and ^&&&^ *P*<0.001, compared with the ACT+FA treatment group.

As an in vitro model for evaluating the neuroprotective effects of ACT and FA, we used CORT‐induced Neuro‐2a cell injury, the results of which revealed that treatment with FA and ACT is associated with significant dose‐dependent reductions in cell viability. At FA and ACT concentrations of 200 and 100 µm, respectively, we detected no cytotoxic effects (**Figure** **6**A,B), and both FA and ACT were found to be effective in protecting N2a cells against CORT‐induced toxicity, with optimal efficacy being observed at 50 µm FA and 25 µm ACT (Figure 6C,D). Interestingly, the protective effects observed in response to the combined treatment with FA (2.5 µm) and ACT (22.5 µm) at a 1:9 ratio were superior to those obtained when either compound was administered alone (Figure 6E,F). In addition, we established that co‐treatment with ACT and FA contributed to restoring the E/I balance disrupted by CORT (Figure 6G,H), thereby confirming their protective effects against CORT‐induced cytotoxicity.

**Figure 7:**
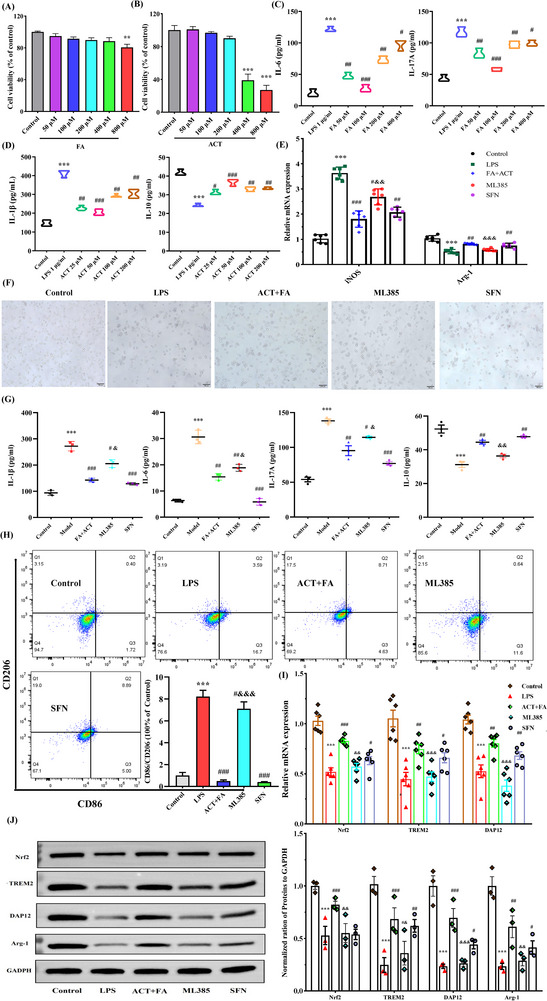
Cooperative effects of ACT and FA on microglial polarization in the LPS‐induced BV2 cells. The effects of FA A) and ACT B) on BV2 cells viability. Effects of FA or ACT single treatments on the levels of IL‐6, IL‐17A C), IL‐1β, and IL‐10 D). E) Neuroprotection of ACT synergistic FA on LPS‐induced BV2 cell morphology (100 µm). F) Statistical analysis of co‐treatment of ACT synergistic FA replicated the equivalent ratio of them in the BDD on the M1 and M2 polarization markers of microglia‐associated mRNAs expression. G) Expression level of the IL‐6, IL‐17A, IL‐1β, and IL‐10 in the supernatant of BV2 cells. H) Flow cytometry of CD86 and CD206 in the LPS‐induced BV2 cells. Quantitative analysis of Nrf2, TREM2, and DAP12 mRNAs I) and proteins J) expression level. GAPDH was set as the internal control. Results are presented as the mean ± SEM of three independent experiments. ^*^ *p*<0.05, ^**^ *p*<0.01 and ^***^ *p*<0.001, compared with the control group; ^#^ *p*<0.05, ^##^ *p*<0.01 and ^###^ *p*<0.001, compared with the LPS‐induced cell model group; ^&^ *P*<0.05, ^&&^ *P*<0.01 and ^&&&^ *P*<0.001, compared with the ACT+FA treatment group.

Having thus established these protective effects, we subsequently examined the effects of ACT and FA on LPS‐induced neuroinflammation and microglial polarization. When used to treat BV2 cells at concentrations between 50 and 400 µm and 50 and 200 µm, FA and ACT, respectively, had no evident detrimental effects on cell viability after 24 h (**Figure** **7**A,B). When administered at 100 µm, FA was found to contribute to a significant enhancement in cell viability, along with a reduction in levels of the pro‐inflammatory cytokines IL‐6 and IL‐17A in LPS‐treated BV2 cells (Figure 7C). Exposure to LPS was found to be associated with a suppression of the M2 marker IL‐10 and a corresponding increase in levels of the M1 marker IL‐1β, thereby indicating a polarization toward the M1 phenotype. These effects were partially reversed by ACT, particularly when administered at 50 µm (Figure 7D). Notably, compared with monotherapies and other combined treatment ratios, treatment with the ACT‐FA (45 µm‐5 µm) combinatorial preparation, formulated according to the original Baihe Dihuang Decoction ratio (ACT: FA = 9:1), was demonstrated to have superior efficacy in synergistically modulating the switching of microglial phenotypes and inflammatory cascades (Figure S7, Supporting Information).

To confirm the role of Nrf2 in mediating the anti‐inflammatory effects of ACT and FA, experiments were repeated using the Nrf2 agonist SFN and inhibitor ML385. Our findings with respect to qRT‐PCR analysis of Arg‐1 and iNOS expression (Figure 7E), BV2 cell morphology (Figure 7F), cytokine levels (Figure 7G), and CD86/CD206 ratios (Figure 7H) consistently revealed that when combined with FA, ACT attenuated microglial activation, thereby promoting a shift from the M1 to M2 phenotype. Whereas inhibition of Nrf2 by ML385 partially abrogated these effects, activation of Nrf2 using SFN was observed to enhance the conversion to an anti‐inflammatory phenotype. Mechanistic studies revealed that whereas exposure to LPS promotes a downregulation of Nrf2, TREM2, DAP12, and Arg‐1 expression in BV2 cells, co‐treatment with ACT and FA reversed these effects (whole uncropped images of the original western blots are shown in Figure S8, Supporting Information). However, this reversal was diminished in the presence of ML385 (Figure 7I,J).

**Figure 8:**
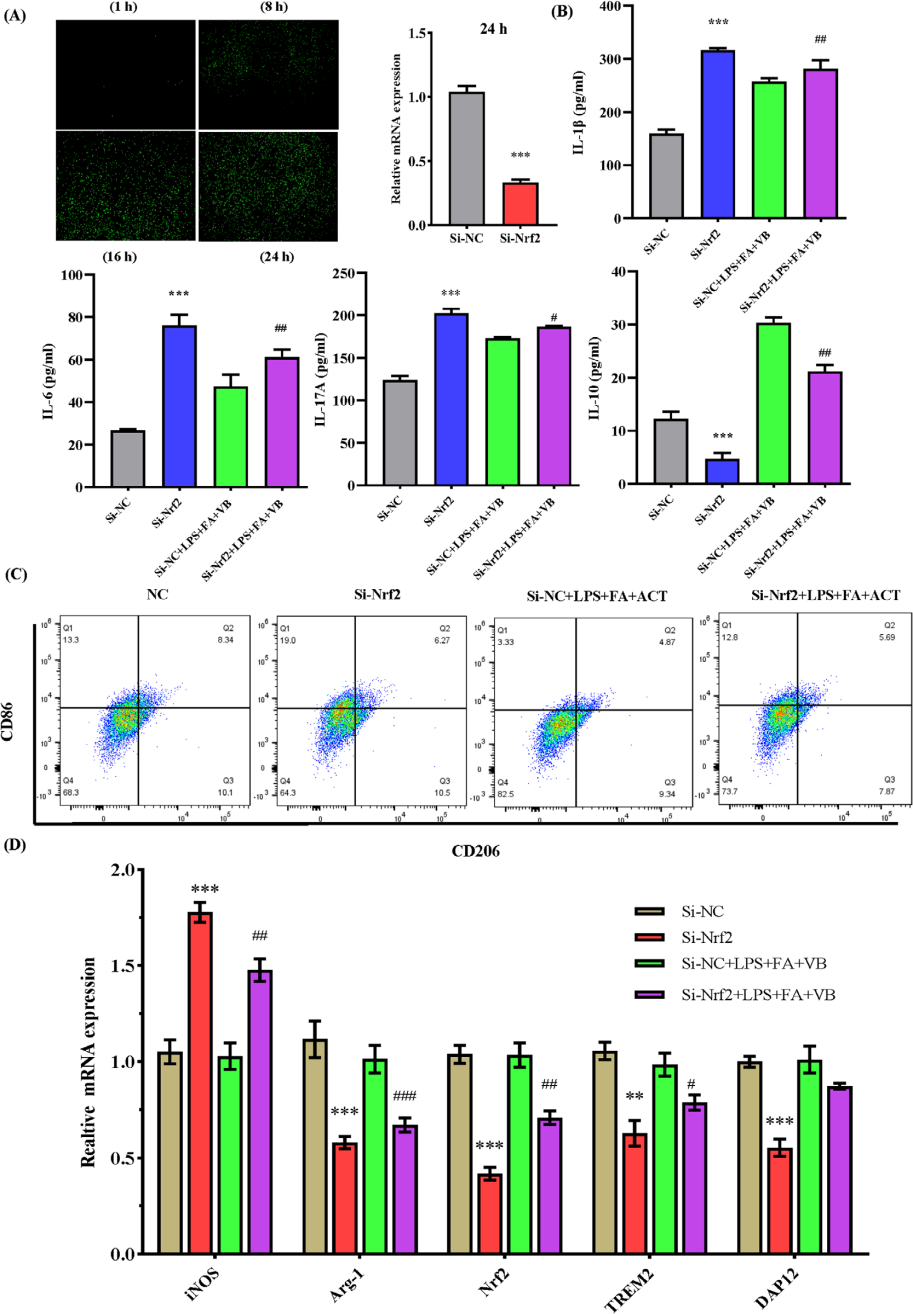
siRNA knockdown of Nrf2 reverses the phenotypic transformation induced by ACT and FA in BV2 cells. A) Schematic diagram of plasmid transfection efficiency with GFP fluorescence in different time points (1/8/16/24 h), and qRT‐PCR was used to detect the knockdown efficiency of siRNA targeted Nrf2. B) The expression levels of IL‐1β, IL‐6, IL‐17A, and IL‐10 in BV2 cells were tested by ELISA. C)The levels of polarization biomarkers of CD86 and CD206 were detected by flow cytograms of BV2 cells in each group. D) Relative mRNA expression levels of M1 and M2 markers, and Nrf2/TREM2/DAP12 in the different groups. Results are presented as the mean ± SEM of three independent experiments. ^*^ *p*<0.05, ^**^ *p*<0.01, and ^***^ *p*<0.001, compared with the Si‐NC group; ^#^ *p*<0.05, ^##^ *p*<0.01, and ^###^ *p*<0.001, compared with the LPS‐induced cell model treated by ACT and FA.

We subsequently investigated whether co‐treatment with ACT and FA retained their efficacy in suppressing the LPS‐induced TREM2/DAP12/Arg‐1 signaling pathways following the siRNA knockdown of Nrf2. Successful knockdown was confirmed by the detection of green fluorescent protein fluorescence and qRT‐PCR analyses (**Figure** **8**A). Moreover, our ELISA results revealed elevated levels of IL‐1β, IL‐6, and IL‐17A following Nrf2 knockdown, whereas those of IL‐10 were reduced (Figure 8B). Correspondingly, we detected an increase in the CD86/CD206 ratio and a reduction in the expression of Arg‐1 mRNA, thereby indicating a shift to the M1 phenotype (Figure 8C,D). Knockdown of Nrf2 was also found to be associated with a downregulation of TREM2/DAP12 signaling, thus counteracting the synergistic anti‐inflammatory effects of ACT and FA (Figure 8B–D). These findings accordingly highlight the pivotal role played by Nrf2 in the cooperative action of ACT and FA in promoting M2 microglial polarization and suppressing microglia‐mediated neuroinflammation via the TREM2/DAP12/Arg‐1 pathway.

## 3 Discussion

The findings of numerous pharmacological and clinical studies have substantiated the efficacy of TCM formulations in alleviating depressive symptoms via multi‐target, multi‐pathway, multi‐level, and multi‐mechanistic modes of action.^[^ ^26^ ^]^ The longstanding and successful application of herbal drug combinations in TCM has motivated our investigation of the synergistic potential of phytochemicals isolated from Chinese Materia medica. Interestingly, it is often found that bioactive compounds with weak or modest efficacy as single agents, or those separated by one or two nodes within a protein‐protein interaction network, are frequently characterized by effective synergistic interactions. In a previous review, the authors similarly posited that the combination strategy and pharmacological synergy between active constituents within medicinal plants offer potential advantages, including fewer side effects and more additive therapeutic benefits in the management of depression.^[^ ^27^, ^28^, ^29^ ^]^ Accordingly, investigating the pharmacological mechanisms of the active constituents in TCM for the management of depression is consistent with modern drug discovery efforts and supports the development of novel antidepressant agents.

Botanical medicines offer a number of therapeutic advantages for the treatment of complex disorders such as depression based on the polypharmacological modulation of multiple targets via diverse components. Synergistic potentiation can be achieved on the basis of both herbal formulations and chemotherapeutic combinations, thereby contributing to reductions in the likelihood of adverse effects. For example, modern research has identified heat‐clearing and depression‐relieving formulations (e.g., BDD and Zhizi Chi Tang) with potent antidepressant activity. However, the phytochemical complexity and incomplete target interaction data currently impede the elucidation of bioactive constituents and their associated mechanisms of action, thereby constraining the systematic investigation of synergistic components. Traditional trial‐and‐error approaches for screening effective component combinations in phytochemical systems are both time‐consuming and costly, thereby limiting their widespread application. Consequently, novel strategies are urgently required to overcome this bottleneck.^[^ ^30^ ^]^ In this regard, recent advances in high‐throughput technologies have generated large‐scale biomedical datasets, and artificial intelligence (AI) and machine learning are increasingly providing ever‐more powerful computational tools for extracting key features from complex systems. Integrating AI with biomedical big data is eagerly anticipated to offer promising solutions for the identification of bioactive constituents and synergistic mechanisms.^[^ ^31^ ^]^ In the present pioneering study, we adopted machine learning and AI‐driven mining of large‐scale biomedical data to predict component‐target interactions and systematically identify synergistic antidepressant phytochemical combinations.

BDD, a co‐decoction of *Lilium brownii* and *Radix Rehmanniae Recens*, is recognized for its pleiotropic effects and has traditionally been used to treat anxiety and depression.^[^ ^32^ ^]^ Over the past 10 years, we have systematically investigated the pharmacodynamic basis of the antidepressant properties of BDD and developed a novel Chinese medicinal formulation based on active constituent compatibility.^[^ ^33^ ^]^ Integrated multi‐source data and machine learning have enabled us to identify ACT and FA as the core bioactive constituents in BDD, the therapeutic targets of which regulate neurotransmitter synthesis and suppress inflammatory responses. Quantitative chemical analyses determined the relative proportions of ACT and FA in BDD, indicating a 9:1 concentration ratio. Interestingly, however, whereas ACT was not detected in juice prepared by crushing fresh *Rehmanniae Radix*, it was detected in boiled fresh *Rehmanniae Radix* juice and BDD, thereby indicating that ACT is produced after heating rather than being an inherent component of fresh *Rehmanniae Radix*.^[^ ^34^, ^35^ ^]^ Similarly, Mao et al. discovered that different processing techniques can have differing effects on the FA content in a Lilii bulbus decoction. In particular, scalding the water‐soaked fresh Lilii bulbs processed by “soaking in water for one night to remove foam” was found to be conducive to the preservation of FA. Furthermore, recent evidence based on bioethnopharmacological analyses indicates that ACT and FA, which can be simultaneously absorbed by the intestinal tract and cerebral cortex of mice, are considered the major absorbed bioactive compounds associated with the therapeutic properties of BDD.^[^ ^36^ ^]^ Consequently, when applied at an equivalent dose ratio as that in BDD, ACT and FA may contribute to the main efficacy of the parent formulation.

Recent studies have identified ACT in a diverse range of Chinese herbs, including *Plantago depressa* Willd., *Cistanche deserticola* Ma, *Olea europaea* L., *Rehmannia glutinosa*, *Verbena officinalis* L., *Lantana camara*, and *Lippia citriodora*, and FA has been detected in *Ligusticum chuanxiong* Hort., *Ferula asafoetida* L., *Ziziphus jujuba* Mill. var. *spinosa*, *Lilium brownii* F.E. Brown var. *vibraculum* Baker, *Angelica sinensis* (Oliv.) Diels, and *Cimicifuga foetida* L. Preparations of these herbs have been shown to have antidepressant effects in both clinical and in vivo studies.^[^ ^37^, ^38^ ^]^ As natural compounds with promising therapeutic applications, ACT and FA have been verified as agents that could be effective in the treatment of depression via different pharmacological mechanisms. Consistent with previous studies, our findings in the present study revealed that the administration of ACT and FA dose‐dependently reversed the CUMS‐induced anxiety/depression‐like behaviors in model mice. Furthermore, the anti‐depressive effects of ACT and FA were found to be comparable to those of fluoxetine and the Japanese Kampo medicine Jia Wei Xiao Yao San. Importantly, we established that the co‐administration of ACT and FA at doses (ACT 45 mg kg^−1^ combined with FA 5 mg kg^−1^) equivalent to the proportional concentration in BDD synergistically replicated the approximate efficacy of the parent BDD formulation in terms of antidepressive effects, which were superior to those of ACT or FA applied alone at the same dosages. These findings thus indicate that when co‐occurring, ACT‐FA interact synergistically to mediate the anti‐depressive activity attributable to BDD. Within the gastrointestinal tract, prior to being absorbed by the bloodstream, ACT undergoes hydrolysis, leading to the formation of several degradation products.^[^ ^39^ ^]^ However, the therapeutic application of FA is hindered by certain unfavorable properties, such as poor water solubility, limited bioavailability, rapid metabolism, and instability under physiological conditions.^[^ ^40^ ^]^ In the present study, ACT and FA were administered via intravenous injection, thereby avoiding passage through the gastrointestinal tract. Furthermore, a combined application may influence the absorption and distribution of the individual components of ACT and FA in the body. ACT and FA can penetrate the BBB and hence reach the brain tissue, thereby enhancing their bioavailability, and thus resulting in synergistic effects. ACT, combined with FA, not only has advantages such as synergistic effects and toxicity reduction for traditional prescriptions with compatibility features but also has a well‐defined composition similar to modern medicines. ACT combined with FA may be a potential therapeutic agent for inflammatory and central nervous system diseases. Thus, multicomponent drugs based on Chinese medicinal formulations are considered an inevitable direction for future research on traditional Chinese medicine prescriptions.

Generally, stress‐induced neuroinflammation, driven by microglial activation, plays a key role in the pathophysiology of depression.^[^ ^41^ ^]^ Upon activation, microglia release inflammatory cytokines and enhance phagocytic activity, causing neuronal injury or conferring protection by controlling the balance between the promotion and suppression of inflammation.^[^ ^42^ ^]^ Accumulating evidence indicates that disrupting microglial hyperactivation could represent an attractive strategy for treating anxiety and depression.^[^ ^43^ ^]^ To verify and build upon these previous findings, we examined the effects of a combination of ACT and FA on microglial phenotypes and accordingly established that the combined administration of ACT and FA promoted an M1 to M2 switch in microglia in the prefrontal cortex of mice with CUMS‐induced depression‐like symptoms and also LPS‐treated BV2 cells, thereby maintaining a balance between the levels of pro‐ and anti‐inflammatory cytokines. In this context, Gomes et al. have previously proposed that ACT can enhance the expression of brain‐derived neurotrophic factors to promote the differentiation of neurons and synapses, and reduce the levels of proinflammatory cytokines such as TNF‐α, IL‐1β, and IL‐6 to alleviate neuroinflammation and neurodegeneration.^[^ ^44^, ^45^ ^]^ It is worth noting in this context that ACT has been established to reduce immune‐inflammatory responses in emotional neural pathways and repair neuronal damage caused by the gut microbiome and gut–brain peptides. In addition, FA has been shown to protect against neuronal cell damage induced by different neurotoxins by inhibiting neuroinflammatory responses and oxidative stress, maintaining calcium homeostasis, and regulating calcium ion channels, thereby rectifying mitochondrial dysfunction and inhibiting apoptosis.^[^ ^46^, ^47^ ^]^ Collectively, these different strands of evidence establish a link between the neuroprotective and anti‐inflammatory effects of ACT and FA. Consequently, we speculate that ACT and FA jointly function to induce a neuroprotective microglial phenotype in the medial prefrontal cortex of chronically stressed mice, possibly via the cooperative regulation of neuroinflammatory immune response signaling pathways.

Nrf2 belongs to the Cap'n’collar (CNC) transcription factor family member, by promoting nuclear translocation, inhibiting ubiquitination degradation, and cytoplasmic Kelch‐like epichlorohydrin‐associated protein 1 (Keap1), which maintains the stability of Nrf2 to inhibit the inflammatory response. Accumulating evidence from clinical and pre‐clinical studies indicates that Nrf2 plays anti‐inflammatory and neuroprotective roles in the pathogenesis of depression.^[^ ^48^, ^49^ ^]^ For example, it has been reported that mice in which Nrf2 has been knocked down are characterized by depression‐like phenotypes, which are associated with increased inflammation and a reduction in the TREM2‐mediated microglial Arg‐1^+^ phenotype.^[^ ^11^, ^50^ ^]^ Moreover, certain Nrf2 activators, such as SFN and dimethyl fumarate, have been found to promote the switch of activated microglial cells from a pro‐inflammatory to an anti‐inflammatory state.^[^ ^51^, ^52^ ^]^ In the present study, we found that ACT and FA act jointly via Nrf2 signaling to induce a neuroprotective microglial phenotype in the prefrontal cortex of chronic stress‐exposed mice and in microglial cells that had been exposed to LPS. Interestingly, in both in vitro and in vivo experiments, we found that ACT and FA administered both alone and combined, promoted significant increases in Nrf2‐induced TREM2 transcription, with their efficacies declining in the order ACT‐FA > ACT alone > FA alone. Furthermore, molecular docking analyses revealed that ACT and FA interact with different sites in the vicinity of the DNA‐binding domain of the Nrf2 protein. Compared with FA, the more negative binding energy and melting curve of ACT indicate the latter's stronger binding to Nrf2, which may explain the differences in the respective efficacies of ACT and FA alone and in combination. However, these effects were abrogated in response to the inhibition of Nrf2 expression via either pharmacological or genetic manipulation. Blocking the Nrf2 signaling pathway abolished the neuroprotective microglia induced by ACT and FA in the prefrontal cortex of CUMS‐exposed mice, as well as the antidepressant effects of the ACT and FA combination. Furthermore, the knockdown of Nrf2 triggered a reduction in microglial TREM2/DAP12 signaling in BV2 cells, with a concomitant exacerbated inflammatory response and M1‐type polarization, thereby negating the therapeutic effects of ACT and FA. These findings are consistent with those of a previous study, in which the authors proposed Nrf2 to be an intermediary molecule in the regulation of microglial function to induce an Arg‐1^+^ microglial phenotype that attenuates the neuroinflammatory response in depression.^[^ ^53^, ^54^ ^]^ We accordingly speculate that ACT and FA have antidepressant and anti‐inflammatory effects associated with the regulation of the microglial phenotype, which is mediated via the Nrf2 signaling pathway.

Chronic environmental stress and traumatic social experiences trigger T helper‐ /microglial‐induced inflammatory responses, leading to neuronal dysfunction and depression‐related behaviors, and recent studies in this regard have shown that pathogenic Th17 cells derived from the abnormal differentiation of prefrontal or peripheral naïve CD4 + T helper cells jointly infiltrate the central nervous system, thereby exacerbating neuroinflammation and depressive symptoms.^[^ ^55^, ^56^ ^]^ Differentiation of Th17 cells from naïve CD4+ T helper cells is dependent on the elevated expression of cytokine IL‐6 or IL‐21, which, in concert with TGF‐β, promotes activation of STAT3 and RORγt.^[^ ^57^ ^]^ In addition, by binding to microglia, astrocytes, and the neuronal surface receptor IL‐17RA, IL‐17A, the main cytokine in Th17 cells, activates and exacerbates immune inflammation‐mediated neuronal damage. Having bound to its microglial receptor, IL‐17A initially recruits nuclear transcription factor KB (NF‐κB) activator 1 (Act1), and the activated Act1 then rapidly recruits TNF‐receptor‐associated factor 6 (TRAF6).^[^ ^58^, ^59^ ^]^ In the present study, we found that inflammatory cytokines, particularly IL‑17A, were upregulated in CUMS‐exposed mice. Interestingly, these changes were accompanied by alterations in IL‐17AR downstream signaling via Act1‐TRAF6‐mediated microglial activation. ACT and FA, when administered in combination at doses equivalent to those in BDD treatment, could reverse neuroinflammation, neuronal damage, and homeostatic alteration in the pathology of the depressive state by inhibiting RORγt‐driven IL‐17A production and promoting the switch of activated microglia from a pro‐inflammatory to an anti‐inflammatory phenotype. These findings indicate that ACT and FA require RORγt and Nrf2 for the induction of neuroprotective microglial cells, and replicate the antidepressant effect of the parent formulation.

As the principal constituent (90% ratio) of a multi‐component Chinese medicine, ACT suppresses central neuroinflammation, which is consistent with the “pathogen‐clearing” principle of TMC, by eliminating endogenous pathogenic heat, whilst concurrently rebalancing excitatory/inhibitory neurotransmitters to embody the “Yin‐Yang harmonization” concept of TMC, reducing oxidative stress and apoptosis, and modulating Th17 differentiation to consolidate therapeutic foundations through multi‐target antidepressant and neuroprotective actions. FA serves as an adjuvant component (10% ratio), pharmacologically complementing ACT by enhancing blood–brain barrier penetration, thereby facilitating access to pathological sites and thus amplifying anti‐inflammatory efficacy. The ACT‐FA combination collaboratively inhibits microglial hyperactivation and promotes M1‐to‐M2 polarization, thereby augmenting anti‐inflammatory outcomes. Maintaining the original 9:1 BDD ratio of Zhang Zhongjing, this precisely calibrated principal constituent‐adjuvant synergy exemplifies traditional Chinese medicinal wisdom, incorporating time‐honored compositional principles to achieve integrated antidepressant, anti‐inflammatory, and neuroprotective effects via cooperative multi‐target modulation.

Recently, self‐assembly nanotechnology has become an increasingly more prominent focus of research.^[^ ^60^ ^]^ On the basis of the direct self‐assembly of active monomers to fabricate carrier‐free nanodrugs, the rate of drug release and half‐life can be precisely regulated.^[^ ^61^ ^]^ In the future, we need to investigate whether self‐assembled nanomaterials are formed between ACT and FA to enhance the antidepressant properties, and elucidate the synergy mechanism underlying the compatibility of ACT and FA against noninflammatory responses to multiple pathogenic brain foci. Upcoming studies will delineate Nrf2 and RORγt‐mediated microglia‐neuron signaling dynamics under ACT‐FA treatment, employing spatial transcriptomics to comparatively evaluate multi‐target versus single‐agent efficacy. Furthermore, the scientific connotation of the formula compatibility theory can be elucidated using multidisciplinary technology empowered by artificial intelligence to verify targets and synergistic molecular mechanisms of ACT and FA.

**Figure 9:**
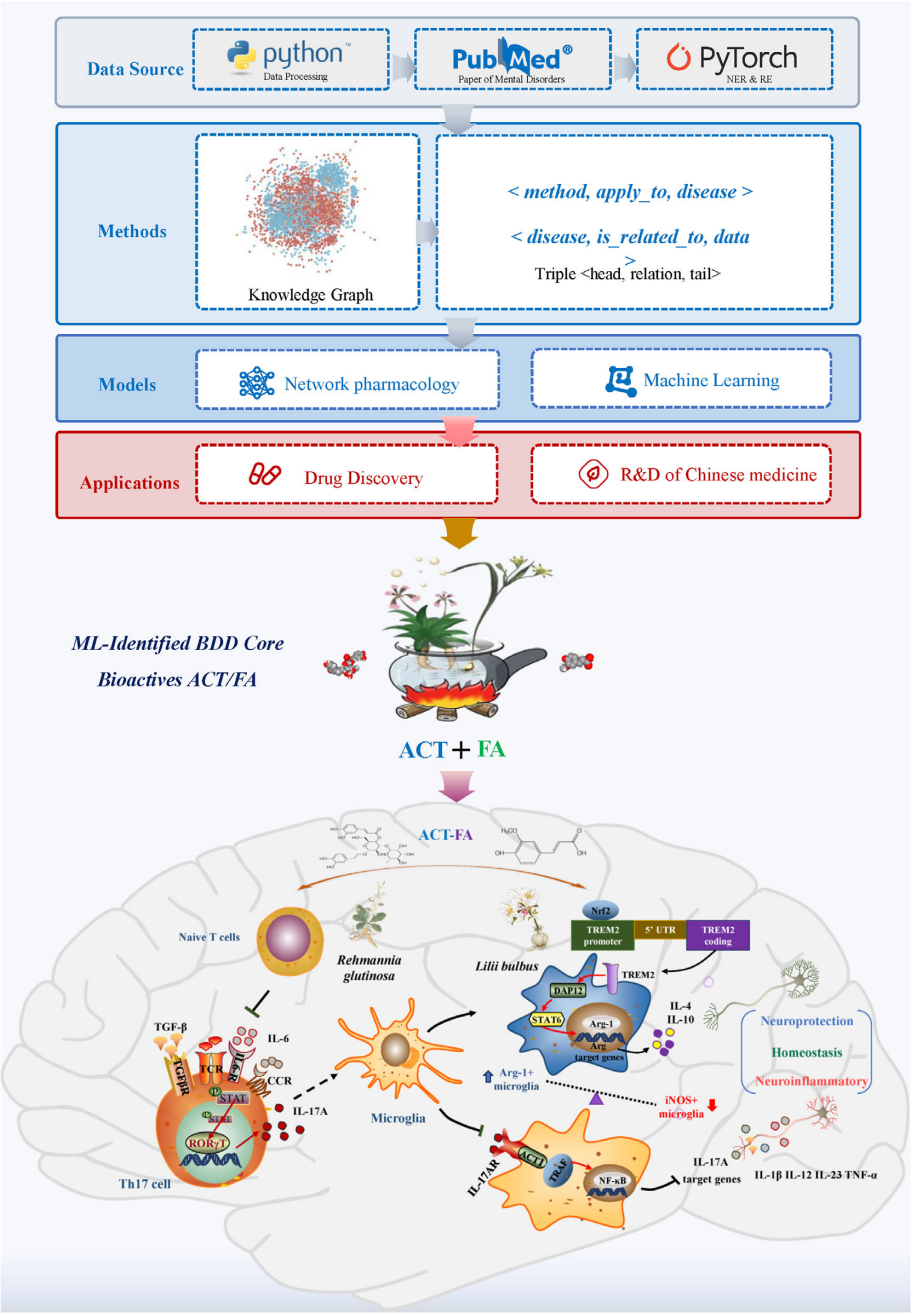
Machine learning‐prioritized BDD components ACT/FA target dual Nrf2/RORγt pathways for microglial therapy in depression. Chronic environmental stress and traumatic social experiences trigger T helper‐/microglial‐ induced inflammatory responses, ultimately leading to neuronal dysfunction and depressive related behavior. Integrated multi‐source data and machine learning identified ACT and FA as core bioactive constituents in BDD, targeting immuno‐inflammatory pathways for depression intervention. Co‐treatment with ACT and FA at an equivalent ratio of them in BDD replicates the neuroprotective antidepressant effects of the parent formula via RORγt coupled with Nrf2‐driven microglia polarization. This dual‐space immunomodulation attenuates neuroinflammation and depressive behaviors by resolving central microglial activation and blocking peripheral inflammatory infiltration, replicating BDD's efficacy to provide a mechanistic basis for multi‐target phytomedicines against depression.

In summary, our findings in this study have revealed that co‐treatment with ACT and FA, at a ratio equivalent to that of each of these compounds in BDD, replicated the antidepressant efficacy of the parent formulation. Mechanistically, ACT and FA synergistically maintained the balance between microglial M1/M2 polarization, effectively attenuating the neuroimmune inflammatory responses. This effect is proposed to be achieved by suppressing the RORγt/IL‐17AR signaling pathway and enhancing the Nrf2/TREM2/DAP12/Arg‐1 pathway, highlighting their cooperative action in mitigating neuroinflammation and depression‐like behaviors (**Figure** **9**). The current study pioneers machine learning and AI‐driven mining of large‐scale biomedical data to predict component‐target interactions and systematically identify synergistic antidepressant phytochemical combinations. Meanwhile, these findings provide a scientific basis for developing ACT and FA as novel antidepressant agents and underscore the therapeutic potential of active ingredients compatibility from traditional Chinese medicine formulations.

## 4 Experimental Section

### Animals and Interventions

Juvenile male C57BL/6J mice (4 weeks old) were purchased from Beijing Vital River Laboratory Animal Technology Co., Ltd (Beijing, China). Following a 7‐day period of acclimation under standard laboratory conditions (12‐h light/dark cycle, 22 ± 1 °C, 50% ± 5% humidity), mice were allocated to control and experimental cohorts using block randomization based on baseline sucrose preference and body weight metrics to ensure intergroup homogeneity prior to intervention. The mice were subsequently housed individually and exposed to a 28‐day CUMS paradigm modified from established protocols.^[^ ^62^ ^]^ All experimental procedures received ethical approval from the Animal Ethics Committee of Shandong University of Traditional Chinese Medicine (Protocol ID: SDUTCM20230629001) in compliance with the ARRIVE and NIH animal use standards.

The BDD formulation was prepared following previously published methods.^[^ ^33^ ^]^ Drug administration commenced 1 week after completing the CUMS protocol. Mice in the BDD group received intragastrically administered BDD (6.25 g kg/day, 0.3 mL) or Jia Wei Xiao Yao San (JWXYS: 0.69 g/kg/day; Kracie, Japan) at the same time each day, along with fluoxetine (Lilly Suzhou Pharmaceutical Co., Ltd., Suzhou, China). Mice in the control and CUMS groups received an equivalent volume of saline (0.3 mL). All interventions were systematically delivered 1 h after daily stressor exposure to minimize circadian interference, with dosing schedules synchronized to ±15 min daily.

After 7 days of exposure to CUMS, mice received a daily intragastric administrations for 21 consecutive days as follows: fluoxetine (Flu: 3 mg kg^−1^; Sichuan Kelun Pharmaceutical Co., Ltd, China); acteoside (ACT: dissolved in 0.9% saline; Nanjing Jingzhu Biotechnology Co., Ltd, China) at graded doses (30, 60, or 90 mg kg^−1^); and ferulic acid (FA): dissolved in 0.9% saline with 1% DMSO; Nanjing Jingzhu Biotechnology Co., Ltd, China) at incremental doses (20, 40, or 80 mg kg^−1^). This pilot treatment phase was designed to establish the optimal FA and ACT concentrations for subsequent experiments. As the final dose of the bioactive compound‐based Chinese herbal formula, an ACT‐to‐FA ratio of 9:1 in BDD was elected, which was consistent with the pharmacodynamic proportions validated in previous research.

To determine the role of Nrf2 in the switching of microglial phenotypes, mice were divided into the following seven experimental groups: control (no treatment), model (CUMS only), FA (CUMS + FA), ACT (CUMS + ACT), ACT+FA (CUMS + combined ACT and FA), ML385 (CUMS + ACT + FA + Nrf2 inhibitor ML385), and SFN (CUMS + Nrf2 activator SFN). SFN (10 mg kg^−1^ dissolved in distilled water containing 10% corn oil; Meryer Chemical Technology Co., Ltd, Shanghai, China) was administered intravenously daily for 21 consecutive days prior to exposure to chronic stress. Mice treated with ACT and FA received ML385 (30 mg/kg/day dissolved in corn oil with 5% dimethyl sulfoxide; Meryer Chemical Technology Co., Ltd, Shanghai, China) via intravenous injection once daily for 3 weeks. SFN and ML385 doses were selected based on previous studies.^[^ ^53^ ^]^ All experimental groups consisted of six to eight mice.

### Network Pharmacology, Machine Learning Algorithms, and Molecular Docking

Given the inherent complexity of the chemical constituents in TCM formulations, UPLC‐MS/MS was employed for high‐throughput compositional analysis of BDD. The chemical structures of the bioactive components of BDD were obtained from DrugBank (https://go.drugbank.com). Potential targets were identified using the Traditional Chinese Medicine Systems Pharmacology Database and Analysis Platform (TCMSP, http://tcmspw.com/tcmsp.php), and subsequent gene symbol standardization was performed using UniProt (https://www.uniprot.org). Depression‐associated targets were systematically curated from three established repositories: DisGeNET (https://www.disgenet.org), GeneCards (https://www.genecards.org), and a Therapeutic Target Database (TTD; https://db.idrblab.net/ttd/). Brain–gut axis transcriptomic data reflecting the antidepressant effects of BDD were obtained from a previously published study.^[^ ^13^ ^]^ Intersection targets were derived from the overlap between common BDD‐depression targets and DEGs identified in the prefrontal cortex transcriptomic data from model depressed mice treated with BDD.

To identify phenotype‐anchored core therapeutic targets for BDD intervention in the treatment of depression, six machine learning algorithms representing complementary methodologies, namely, least absolute shrinkage and selection operator (LASSO) were strategically deployed, random forest (RF), extreme gradient boosting (XGBoost), Boruta, support vector machine (SVM), and Bayesian. Genes consistently identified when using all algorithms were designated final core therapeutic targets for BDD‐based depression interventions. For further validation, the Rms package was employed to generate bar plots for candidate therapeutic markers and the pROC package to compute ROC‐derived area under the curve (AUC) values to quantitatively assess discriminatory performance.

The intersections of the targets associated with ACT and FA and those linked to depression were identified as core targets for further analysis. Using these cire targets, Kyoto Encyclopedia of Genes and Genomes (KEGG) pathway enrichment analysis was performed and constructed protein–protein interaction (PPI) networks, with Sankey diagrams being generated to visualize the component–target‐pathway network. The 3D crystal structures of the core targets were obtained from the Protein Data Bank (PDB) database. Molecular docking was conducted using AutoDock Vina (https://vina.scripps.edu/), with results showing the highest docking scores and optimal conformations being selected as the final molecular docking outcomes.

### Behavioral Tests and Enzyme‐Linked Immunosorbent Assays

The sucrose preference, forced swimming, open field, and elevated plus maze tests were performed to measure depression‐like behavior, as previously described.^[^ ^63^ ^]^ Following the final intervention, the mice were decapitated, and blood samples were collected using vacuum tubes. The samples were centrifuged for 15 min at 4 °C and 3000 rpm, and the serum thus obtained was stored at ‐80 °C for subsequent analysis. Commercial ELISA kits (Jing Mei Biotech, Yancheng, China) were used to measure the serum levels of interleukin (IL)‐1β, IL‐6, IL‐17A, and IL‐10, as well as the neurotransmitters γ‐aminobutyric acid (GABA) and glutamic acid. Absorbances were measured at 450 and 570 nm using an ELx808 microplate reader (Biotek, USA).

### Cell Culture, Treatment, and Transfection

Neuro‐2a (N2a) cells, obtained from the Kunming Institute of Zoology, Chinese Academy of Sciences, were plated in Dulbecco's modified Eagle's medium (DMEM; Corning, New York, USA) supplemented with 10% fetal bovine serum (FBS) and 1% penicillin–streptomycin. The cells were initially exposed to corticosterone (CORT: 600 µm; Sigma‐Aldrich, Missouri, USA) for 24 h and subsequently treated with FA (25, 50, 100, or 200 µm) or ACT (12.5, 25, 50, or 100 µm). Mifepristone (2.5 µm; RU486), a CORT receptor antagonist, served as a positive control. To determine the optimal concentrations of FA and ACT for mitigating CORT‐induced neuronal damage, cell viability was assessed using a CCK‐8 assay kit (Dojindo Laboratories).

To exclusively investigate central neuroimmunomodulatory mechanisms without peripheral interference, a BV‐2 microglial model was used, which enabled us to obtain a cell‐based validation of target engagement and signaling pathway regulation.^[^ ^64^ ^]^ BV‐2 microglial cells were pre‐treated with FA (50100, 200 and 400 µm), ACT (25, 50100 and 200 µm), or the positive control drug minocycline (100 µm) for 30 min, followed by the addition of PBS or lipopolysaccharide (LPS: 1 µg mL^−1^; Solaibao Biotechnology Co., Ltd, Beijing China) for 24 h. To determine the effective concentrations of FA and ACT for alleviating neuroinflammation, the levels of IL‐1β, IL‐6, IL‐17A, and IL‐10 in culture supernatants were determined, which were quantified using commercial ELISA kits (Jing Mei Biotech, Yancheng, China). The dose‐response relationships of ACT/FA co‐treatment (at ACT:FA ratios of 1:9, 1:1, and 9:1) and positive control minocycline (Mino; 1 µmol/L) were systematically evaluated in BV‐2 cells using flow cytometric analysis of microglial polarization markers and ELISA quantification of inflammatory factors. For further analysis, LPS‐primed BV2 cells were treated with a combination of ACT and FA, ML385 (10 µm), or SFN (1 µm). The experimental groups included control, LPS, LPS + ACT + FA, LPS + ACT + FA + ML385, and LPS + SFN groups.

Small interfering RNA (siRNA) targeting Nrf2 (Shandong Keyybio Biotechnology Co., China) was used to silence Nrf2 mRNA expression in BV‐2 cells, using the interference sequences listed in Table S5 (Supporting Information), and off‐target effects were evaluated using a negative control (NC) sequence. Transfection was performed using a Lipofectamine 3000 kit (Invitrogen, Thermo Fisher Scientific, Massachusetts, USA). siRNA diluted in Opti‐MEM was added to the complete medium without FBS and introduced into the wells containing the cells. After 24 h, the medium was replaced with DMEM containing 10% FBS, followed by LPS treatment and exposure to FA or ACT for 24 h. For siRNA‐based experiments, the following five treatments were assessed: control, Si‐NC, Si‐Nrf2, Si‐NC + LPS + ACT + FA, and Si‐Nrf2 + LPS + ACT + FA.

### Flow Cytometric Analysis

The proportions of Th17/CD4‐positive cells (IL‐17A and GA1177, Servicebio; CD4 and PE‐65104, Proteintech) in the brain tissues of mice were determined by flow cytometry. For microglial polarization phenotype analysis, BV‐2 cells were harvested 24 h after treatment, washed sequentially in ice‐cold flow buffer, and incubated with specific antibodies against CD206 (bsm‐30276A‐FITC; Bioss) and CD86 (bsm‐30162A‐PE; Bioss). The samples were analyzed using a CytoFlex flow cytometer (Beckman Coulter), and data were further processed using FlowJo Software (Tree Star Inc., Ashland, OR, USA).

### Immunofluorescence Staining

Having removed the entire brains from decapitated mice, these were then fixed in 4% paraformaldehyde fix solution overnight at 4 °C, and following subsequent dehydration and embedding in paraffin, the mPFC tissues were cut into 5‐µm‐thick coronal sections. For immunofluorescence staining, following dehydration, antigen repair, and blocking with 2% BSA, sections were incubated overnight at 4 °C with 20 µL of RORγt (DF3196; Affinity), IBA‐1 (DF6442; Affinity), IL‐17AR (GB11586; Servicebio), ACT1 (26692‐1‐AP; Proteintech), or TRAF6 (GB112201; Servicebio) antibodies. Following three subsequent PBS rinses, the sections were incubated with fluorescent secondary antibodies (A32723; Thermo Fisher Scientific) for 30 min at room temperature. Nuclei were stained with DAPI, and fluorescence signals were observed and images obtained using a Nikon Eclipse Ci‐L fluorescence microscope (Nikon, Japan). Quantitative analysis was performed using Image‐Pro Plus 6 software (Media Cybernetics).

### Cellular Thermal Shift Assay

BV2 microglial cells were lysed in lysis buffer supplemented with protease and phosphatase inhibitors. Soluble proteins were separated by centrifugation for 15 min at 12 000 × *g* and 4 °C. The resulting supernatants were divided into three equal parts, which were respectively incubated with ACT (50 µm), FA (100 µm), or DMSO at 4 °C for 1 h. Each sample was divided into eight aliquots, heated at 35, 40, 45, 50, 55, 60, 65, or 70 °C for 3 min, and centrifuged again to collect the soluble fraction. The resulting supernatants were prepared in a loading buffer and analyzed using western blotting over three cycles.

### Quantitative RT‐PCR and Western Blotting

Total RNA was isolated using TRIzol reagent, followed by cDNA synthesis using PrimeScript RT reagent kits (RR047A; Takara, Liaoning, China). Amplification was performed using a Power SYBR Green Premix (Takara) in conjunction with an ABI 7500 System (Applied Biosystems, California, USA). Levels of the amplified mRNAs relative to controls were determined using the 2^−ΔΔCt^ method, with the *GAPDH* gene serving as an internal control for normalization of the qRT‐PCR data. The sequences of the primers used for amplification were listed in Table S5 (Supporting Information). For western blotting, total proteins from the mPFC tissues were extracted using an Extraction Kit (Beyotime Biotechnology, Shanghai, China). After blocking with 5% skimmed milk, membranes were incubated overnight at 4 °C with primary antibodies against RORγt (DF3196; Affinity), IBA‐1 (DF6442; Affinity), IL‐17AR (GB11586; Servicebio), Act1 (26692‐1‐AP; Proteintech), TRAF6 (GB112201; Servicebio), Nrf2 (GB115673; Servicebio), TREM2 (DF12529; Affinity), DAP12 (DF7316; Affinity), Arg‐1 (GB11285; Servicebio), or GAPDH (1:10000, GB15004; Servicebio). The levels of protein expression were visualized using a Gel Doc XR+ imaging system (Bio‐Rad, Hercules, California, USA) and quantified using ImageJ software.

### Statistical Analysis

Experimental results were presented as the means ± standard error of the mean (SEM). Statistical comparisons between two groups were performed using unpaired *t*‐tests, whereas one‐way ANOVA followed by Tukey's post hoc test was performed for analyses among groups. In all cases, statistical significance was defined as a *p*‐value < 0.05. Statistical analysis was carried out using SPSS 20.0 software (IBM, Armonk, USA).

### Abbreviation

Acteoside, ACT; Artificial intelligence, AI; A small interfering RNA, siRNA; Area Under Curve, AUC; Arginase 1, Arg‐1; Blood brain barrier, BBB; Cellular thermal shift assay, CETSA; Baihe Dihuang Tang, BDD; Corticosterone, CORT; Central nervous system, CNS; Chronic unpredictable mild stress, CUMS; Differentially expressed genes, DEGs; DNAX activation protein of 12 kDa, DAP12; Excitation/inhibition, E/I; Enzyme‐linked immunosorbent assay, ELISA; Elevated plus maze test, EPMT; Ferulic Acid, FA; Forced swimming test, FST; Fetal bovine serum, FBS; Glutamic acid, Glu; Inducible nitric oxide synthase, iNOS; Kyoto Encyclopedia of Genes and Genomes, KEGG; Least Absolute Shrinkage and Selection Operator, LASSO; Lipopolysaccharide, LPS; medial prefrontal cortex, mPFC; Microglia, MG; NAc; nucleus accumbens; Nuclear factor erythroid 2‐related factor 2, Nrf2; Neuro‐2a, N2a; Open field test, OFT; One‐way analysis of variance, ANOVA; Protein‐protein interaction, PPI; Retinoid‐related orphan receptor gamma t, RORγt; Random Forest, RF; Sucrose preference test, SPT; Support Vector Machine, SVM; γ‐aminobutyric acid, GABA; T helper 17, Th17; Triggering receptor expressed on myeloid cell‐2, TREM2; Traditional Chinese medicine, TCM; Traditional Chinese medicine systems pharmacology database and analysis, TCMSP; Protein Data Bank, PDB; TNF‐receptor‐associated factor 6, TRAF6; Western blotting, WB; eXtreme Gradient Boosting, XGBoost.

## Conflict of Interest

The authors declare no conflicts of interest related to this work.

## Author Contributions

D.G., Q.M., and X.F. contributed equally to this work. K.M. designed the research and revised the manuscript. D.G. and K.M. wrote the manuscript. L.H., F. Z., and H.T. carried out the experiments and performed data analysis. W.Y., W.P., and X.F. gave methodological support and conceptual advice. All of the authors have read and approved the final manuscript.

## Supporting information



Supporting Information

## Data Availability

All data needed for evaluating the conclusions in this report can be found in the paper and/or in the Supplementary Materials.

## References

[1] M. E. Fox , M. K. Lobo , Mol. Psychiatry 2019, 24, 1798. 30967681 10.1038/s41380-019-0415-3 PMC6785351

[2] J. Henssler , Y. Schmidt , U. Schmidt , G. Schwarzer , T. Bschor , C. Baethge , Lancet Psychiatry 2024, 11, 526. 38851198 10.1016/S2215-0366(24)00133-0

[3] A. G. Kokkosis , M. M. Madeira , Z. Hage , K. Valais , D. Koliatsis , E. Resutov , S. E. Tsirka , Glia 2024, 72, 111. 37675659 10.1002/glia.24464 PMC10842267

[4] X. Zhong , S. Gong , L. Meng , W. Yao , K. Du , L. Jiao , G. Ma , J. Liang , B. Wei , X. Jin , J. Tong , J. Dong , M. Liu , M. Gao , H. Jia , W. Jiang , Z. Yu , Y. Wang , X. Sun , M. Wei , M. Liu , Adv. Sci. 2024, 11, 2304687. 10.1002/advs.202304687 PMC11336950 38889331

[5] H. Zhu , A. Guan , J. Liu , L. Peng , Z. Zhang , S. Wang , J. Neuroinflammation 2023, 20, 223. 37794488 10.1186/s12974-023-02901-y PMC10548593

[6] E. Beurel , E. M. Medina‐Rodriguez , R. S. Jope , Pharmacol. Rev. 2022, 74, 373. 35302045 10.1124/pharmrev.120.000256 PMC8973514

[7] J. Kim , Y. H. Suh , K. A. Chang , Mol. Brain 2021, 14, 11. 33441182 10.1186/s13041-020-00726-x PMC7805143

[8] Z. Peng , S. Peng , K. Lin , B. Zhao , L. Wei , Q. Tuo , D. Liao , T. Yuan , Z. Shi , J. Neuroinflammation 2022, 19, 186. 35836182 10.1186/s12974-022-02543-6 PMC9281140

[9] K. Alves de Lima , J. Rustenhoven , S. Da Mesquita , M. Wall , A. F. Salvador , I. Smirnov , G. Martelossi Cebinelli , T. Mamuladze , W. Baker , Z. Papadopoulos , M. B. Lopes , W. S. Cao , X. S. Xie , J. Herz , J. Kipnis , Nat. Immunol. 2020, 21, 1421. 32929273 10.1038/s41590-020-0776-4 PMC8496952

[10] C. Zuo , H. Cao , Y. Song , Z. Gu , Y. Huang , Y. Yang , J. Miao , L. Zhu , J. Chen , Y. Jiang , F. Wang , Redox Biol. 2022, 58, 102522. 36335763 10.1016/j.redox.2022.102522 PMC9641011

[11] L. He , Y. Zheng , L. Huang , J. Ye , Y. Ye , H. Luo , X. Chen , W. Yao , J. Chen , J. C. Zhang , Transl. Psychiatry 2022, 12, 459. 36316319 10.1038/s41398-022-02227-y PMC9622811

[12] W. Pan , H. Shi , Z. Zang , Q. Meng , Y. Cheng , L. Liang , Y. Zhai , G. Yin , L. Sun , K. Ma , Heliyon 2024, 10, 25171. 10.1016/j.heliyon.2024.e25171 PMC10862512 38352746

[13] Q. Mao , H. Zhang , Z. Zhang , Y. Lu , J. Pan , D. Guo , L. Huang , H. Tian , K. Ma , Phytomedicine 2024, 129, 155510. 38696921 10.1016/j.phymed.2024.155510

[14] X. Xue , J. Pan , H. Zhang , Y. Lu , Q. Mao , K. Ma , J. Ethnopharmacol. 2022, 292, 115218. 35337919 10.1016/j.jep.2022.115218

[15] H. Zhang , X. Xue , J. Pan , X. Song , X. Chang , Q. Mao , Y. Lu , H. Zhao , Y. Wang , X. Chi , S. Wang , K. Ma , Chin. Med. 2021, 16, 107. 34674715 10.1186/s13020-021-00519-x PMC8529377

[16] X. Dong , R. Huang , Phytomedicine 2022, 105, 154355. 35908520 10.1016/j.phymed.2022.154355

[17] X. Dong , D. Zhao , CNS Neurosci Ther. 2023, 29, 2397. 37183361 10.1111/cns.14265 PMC10401106

[18] M. Sabti , K. Sasaki , C. Gadhi , H. Isoda , Int. J. Mol. Sci. 2019, 20, 3556. 31330819 10.3390/ijms20143556 PMC6678442

[19] L. Deng , X. Zhou , G. Tao , W. Hao , L. Wang , Z. Lan , Y. Song , M. Wu , J. Q. Huang , Food Res. Int. 2022, 162, 111887. 36461269 10.1016/j.foodres.2022.111887

[20] Y. Xiao , Q. Ren , L. Wu , Biomed. Pharmacother. 2022, 153, 113296. 35724511 10.1016/j.biopha.2022.113296 PMC9212779

[21] Y. Xiang , M. Li , E. Pan , Y. Li , W. Yan , Y. Li , G. Ji , J. Dong , Fish Shellfish Immunol. 2024, 151, 109659. 38797333 10.1016/j.fsi.2024.109659

[22] M. Farhan , Z. Saeed , S. Rehman , A. Bibi , S. R. Zehra , S. Ahmed , S. Asif , A. A. Soomro , Pak. J. Pharm. Sci. 2023, 36, 1389. 37869914

[23] Y. Zhao , S. Wang , J. Pan , K. Ma , Phytomedicine 2023, 120, 155027. 37657207 10.1016/j.phymed.2023.155027

[24] T. Jung , C. Cheon , Integr. Cancer Ther. 2024, 23, 15347354241259416. 38867515 10.1177/15347354241259416 PMC11179546

[25] A. Panossian , T. Lemerond , T. Efferth , Pharmaceuticals 2024, 17, 483. 38675443 10.3390/ph17040483 PMC11053582

[26] S. Lv , N. Yang , Y. Lu , G. Zhang , X. Zhong , Y. Cui , Y. Huang , J. Teng , Y. Sai , Front. Pharmacol. 2024, 15, 1426769. 39253375 10.3389/fphar.2024.1426769 PMC11381291

[27] F. Bi , Z. Wang , Y. Guo , M. Xia , X. Zhu , W. Qiao , Curr. Drug Metab. 2024, 25, 71. 38415474 10.2174/0113892002284230240213064248

[28] W. Dai , K. Feng , X. Sun , L. Xu , S. Wu , K. Rahmand , D. Jia , T. Han , J. Ethnopharmacol. 2022, 285, 114692. 34742864 10.1016/j.jep.2021.114692

[29] X. Liu , M. Luo , Z. Wang , S. J. Yang , M. Su , Y. Wang , W. Wang , Z. Sun , Y. Cai , L. Wu , R. Zhou , M. Xu , Q. Zhao , L. Chen , W. Zuo , Y. Huang , P. Ren , X. Huang , Phytomedicine 2024, 128, 155324. 38552437 10.1016/j.phymed.2023.155324

[30] K. Zhang , X. Yang , Y. Wang , Y. Yu , N. Huang , G. Li , X. Li , J. C. Wu , S. Yang , Nat. Med. 2025, 31, 45. 39833407 10.1038/s41591-024-03434-4

[31] S. Gao , A. Fang , Y. Huang , V. Giunchiglia , A. Noori , J. R. Schwarz , Y. Ektefaie , J. Kondic , M. Zitnik , Cell 2024, 187, 6125. 39486399 10.1016/j.cell.2024.09.022

[32] H. Wu , R. Liu , J. Wang , T. Li , Y. Sun , X. Feng , Y. Bi , C. Zhang , Y. Sun , J. Sep. Sci. 2021, 44, 3933. 34473407 10.1002/jssc.202100434

[33] X. Chi , S. Wang , Z. Baloch , H. Zhang , X. Li , Z. Zhang , H. Zhang , Z. Dong , Y. Lu , H. Yu , K. Ma , Biomed. Pharmacother. 2019, 112, 108616. 30780102 10.1016/j.biopha.2019.108616

[34] L. Tang , H. Q. Zhao , H. Yang , C. Hu , S. J. Ma , W. Z. Xiao , Y. H. Qing , L. Yang , R. R. Zhou , J. Liu , S. H. Zhang , J. Ethnopharmacol. 2024, 319, 117090. 37640258 10.1016/j.jep.2023.117090

[35] M. Wei , F. Chen , Z. Zhang , Y. Zhao , R. Li , B. Li , Q. He , Chin. J. Exp. Tradit. Med. Formulae. 2022, 28, 133.

[36] J. Pan , Y. Lu , S. Wang , T. Ma , X. Xue , Z. Zhang , Q. Mao , D. Guo , K. Ma , Phytomedicine 2023, 121, 155102. 37748389 10.1016/j.phymed.2023.155102

[37] C. Fu , Q. Shuang , Y. Liu , L. Zeng , W. Su , ACS Chem. Neurosci. 2022, 13, 587. 35139304 10.1021/acschemneuro.1c00439

[38] X. Zhang , C. Wang , Stud. Health Technol. Inform. 2023, 308, 417. 38007768 10.3233/SHTI230868

[39] Y. Yang , D. Xi , Y. Wu , T. Liu , Plant Commun. 2023, 4, 100592. 36935606 10.1016/j.xplc.2023.100592 PMC10363510

[40] J. R. Purushothaman , M. Rizwanullah , Cureus 2024, 16, 68063. 10.7759/cureus.68063 PMC11438535 39347187

[41] W. J. Su , J. M. Li , T. Zhang , Z. Y. Cao , T. Hu , S. Y. Zhong , Z. Y. Xu , H. Gong , C. L. Jiang , Prog. Neuropsychopharmacol Biol. Psychiatry 2023, 126, 110796. 37209992 10.1016/j.pnpbp.2023.110796

[42] B. Li , W. Yang , T. Ge , Y. Wang , R. Cui , Pharmacol. Res. 2022, 179, 106145. 35219870 10.1016/j.phrs.2022.106145

[43] X. Guo , Y. Rao , R. Mao , L. Cui , Y. Fang , Neuropharmacology 2020, 181, 108336. 32980387 10.1016/j.neuropharm.2020.108336

[44] D. B. Gomes , P. Z. Serpa , D. Miorando , M. Zanatta , C. S. Carteri , L. B. Somensi , L. Venzon , A. C. Santos , T. C. S. Franca , L. M. Silva , W. A. Roman Junior , Evid Based Complement Alternat. Med. 2022, 2022, 1041656. 36185078 10.1155/2022/1041656 PMC9522501

[45] G. L. Viswanatha , H. Shylaja , D. V. Kishore , M. V. Venkataranganna , N. B. L. Prasad , Neurotox. Res. 2020, 38, 1010. 32803629 10.1007/s12640-020-00267-0

[46] X. Chen , X. Zhou , X. Cheng , L. Lin , Q. Wang , R. Zhan , Q. Wu , S. Liu , Molecules 2023, 28, 3482. 37110714 10.3390/molecules28083482 PMC10217031

[47] X. Zhou , X. Chen , X. Cheng , L. Lin , S. Quan , S. Li , R. Zhan , Q. Wu , S. Liu , J. Pharmacol. Sci. 2023, 152, 151. 37169480 10.1016/j.jphs.2023.04.007

[48] R. Subba , M. H. Ahmad , B. Ghosh , A. C. Mondal , Eur. J. Pharmacol. 2022, 925, 174993. 35513015 10.1016/j.ejphar.2022.174993

[49] G. Sani , S. Margoni , A. Brugnami , O. M. Ferrara , E. Bernardi , A. Simonetti , L. Monti , M. Mazza , D. Janiri , L. Moccia , G. D. Kotzalidis , D. P. R. Chieffo , L. Janiri , Antioxidants 2023, 12, 817. 37107192 10.3390/antiox12040817 PMC10135298

[50] F. D. Shi , V. W. Yong , Science 2025, 388, adx0043. 10.1126/science.adx0043 40536983

[51] P. Panczyszyn‐Trzewik , K. Stachowicz , P. Misztak , G. Nowak , M. Sowa‐Kucma , Pharmaceuticals 2024, 17, 762. 38931429 10.3390/ph17060762 PMC11206991

[52] R. Tang , Q. Q. Cao , S. W. Hu , L. J. He , P. F. Du , G. Chen , R. Fu , F. Xiao , Y. R. Sun , J. C. Zhang , Q. Qi , Acta Pharmacol. Sin. 2022, 43, 829. 34272506 10.1038/s41401-021-00727-z PMC8976037

[53] J. Zhang , L. Li , Q. Liu , Z. Zhao , D. Su , C. Xiao , T. Jin , L. Chen , C. Xu , Z. You , T. Zhou , Phytomedicine 2023, 113, 154725. 36867963 10.1016/j.phymed.2023.154725

[54] A. Nellessen , S. Nyamoya , A. Zendedel , A. Slowik , C. Wruck , C. Beyer , A. Fragoulis , T. Clarner , Metab. Brain Dis. 2020, 35, 353. 31529356 10.1007/s11011-019-00488-z

[55] E. M. Medina‐Rodriguez , J. Watson , J. Reyes , M. Trivedi , E. Beurel , Microbiome 2023, 11, 92. 37106375 10.1186/s40168-022-01428-3 PMC10142784

[56] S. Inan , J. J. Meissler , S. Bessho , S. Wiah , C. Tukel , T. K. Eisenstein , S. M. Rawls , Brain Behav. Immun. 2024, 117, 100. 38199516 10.1016/j.bbi.2024.01.001 PMC10932873

[57] H. Wang , Y. He , Z. Sun , S. Ren , M. Liu , G. Wang , J. Yang , J. Neuroinflammation 2022, 19, 132. 35668399 10.1186/s12974-022-02492-0 PMC9168645

[58] W. Lin , N. Wang , K. Zhou , F. Su , Y. Jiang , J. Shou , H. Liu , C. Ma , Y. Qian , K. Wang , X. Wang , EMBO Rep. 2019, 20, 47502. 10.15252/embr.201847502 PMC6362353 30723107

[59] A. M. Mustafa , A. M. Shaheen , H. F. Zaki , M. A. Rabie , Int. Immunopharmacol. 2024, 127, 111387. 38134593 10.1016/j.intimp.2023.111387

[60] X. Guo , W. Luo , L. Wu , L. Zhang , Y. Chen , T. Li , H. Li , W. Zhang , Y. Liu , J. Zheng , Y. Wang , Adv. Sci. 2024, 11, 2403388. 10.1002/advs.202403388 PMC11425287 39033533

[61] K. Ge , Z. Li , A. Wang , Z. Bai , X. Zhang , X. Zheng , Z. Liu , F. Gao , ACS Nano 2023, 17, 2222. 36688477 10.1021/acsnano.2c08499

[62] K. Ma , A. Xu , S. Cui , M. R. Sun , Y. C. Xue , J. H. Wang , Transl. Psychiatry 2016, 6, 910. 10.1038/tp.2016.181 PMC5315548 27701406

[63] X. Tian , G. Wang , F. Teng , X. Xue , J. Pan , Q. Mao , D. Guo , X. Song , K. Ma , CNS Neurosci Ther. 2023, 30, 14519. 10.1111/cns.14519 PMC11017446 37905694

[64] J. Cheng , R. Zhang , Z. Xu , Y. Ke , R. Sun , H. Yang , X. Zhang , X. Zhen , L. T. Zheng , J. Neuroinflammation 2021, 18, 129. 34107997 10.1186/s12974-021-02187-y PMC8191212

