# Supplementary material for: Synergistic Modulation of Microglial Polarization by Acteoside and Ferulic Acid via Dual Targeting of Nrf2 and RORγt to Alleviate Depression‐Associated Neuroinflammation
Source: Adv Sci (Weinh). 2025 Aug 20;12(43):e03889. doi: 10.1002/advs.202503889 (PMC12631924; doi:10.1002/advs.202503889)
Supplement: Supplementary file 1 — Supporting Information [file ADVS-12-e03889-s001.docx]

**Synergistic Modulation of Microglial Polarization by Acteoside and Ferulic Acid via Dual Targeting of Nrf2 and RORγt to Alleviate Depression-associated Neuroinflammation**

Dongjing Guo ^#^, Qiancheng Mao ^#^, Xinyu Fang ^#^, Liuxuan Huang, Haoquan Tian, Wenguang Yang, Feiyue Zhou, Ke Ma *

*Shandong Co-Innovation Center of Classic TCM Formula, Shandong University of Traditional Chinese Medicine, Jinan 250355, PR China*

**Running title:** Synergistic neuroprotective effects of ACT and FA.

# These authors contributed equally to this work

**Address Correspondence to:**

Dr. Ke Ma, Shandong Co-Innovation Center of Classic TCM Formula, Shandong University of Traditional Chinese Medicine, No 4655, University Road, Changqing District, Jinan, Shandong 250355, China, Tel/Fax +86-531-89628077, Email: make19880710@163.com.

**Figure S1: Comparison of different concentrations of BDD antidepressant effects.**

**
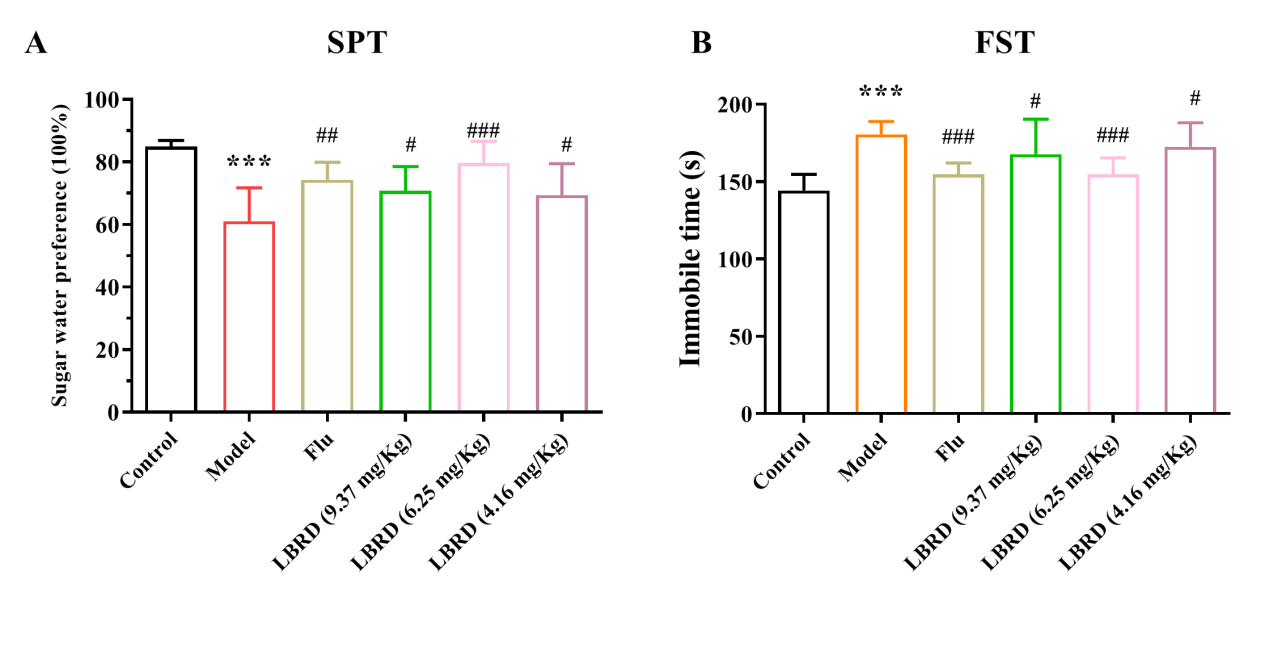
**

**Figure S2: The pattern plot of the protein band in Figure 2I is derived from the whole un-cropped images of the original western blots.**


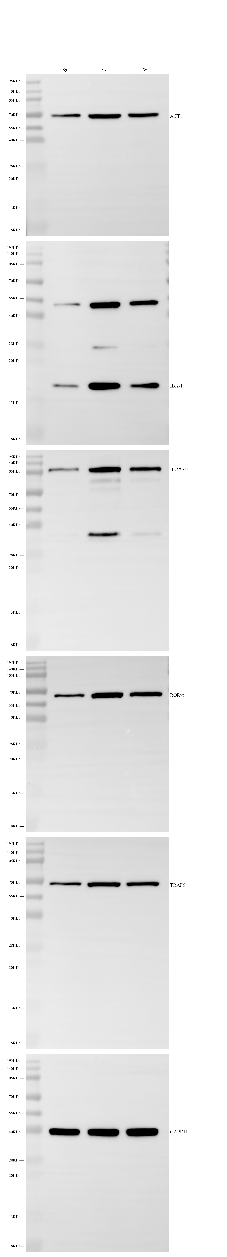

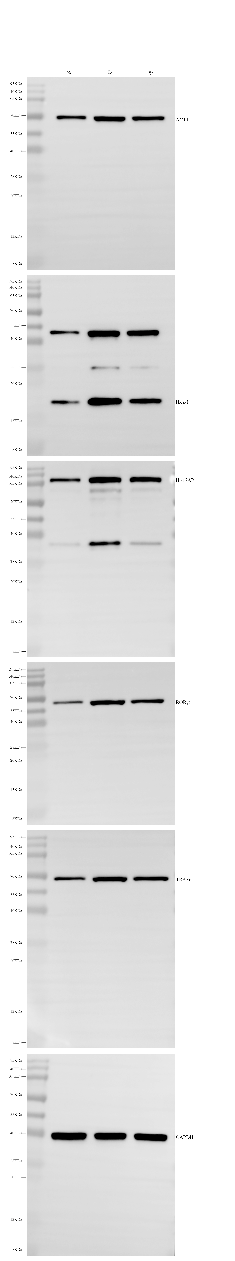

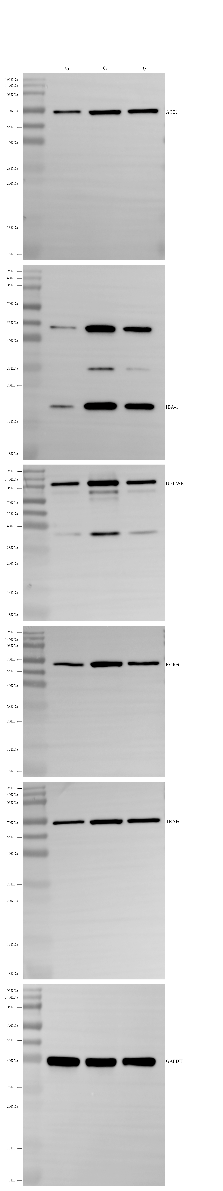


**Sample 1 Sample 2 Sample 3**


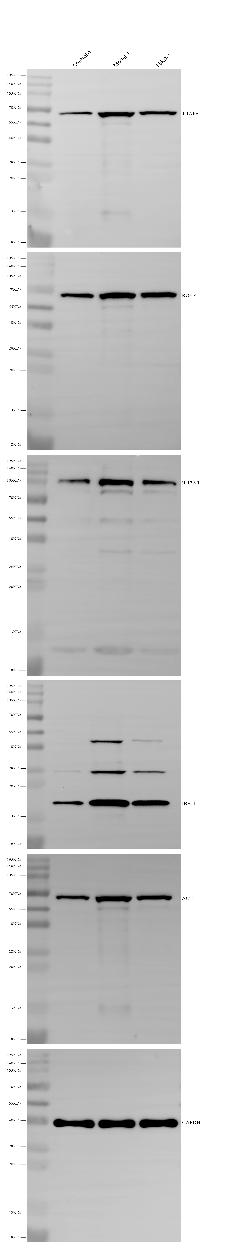

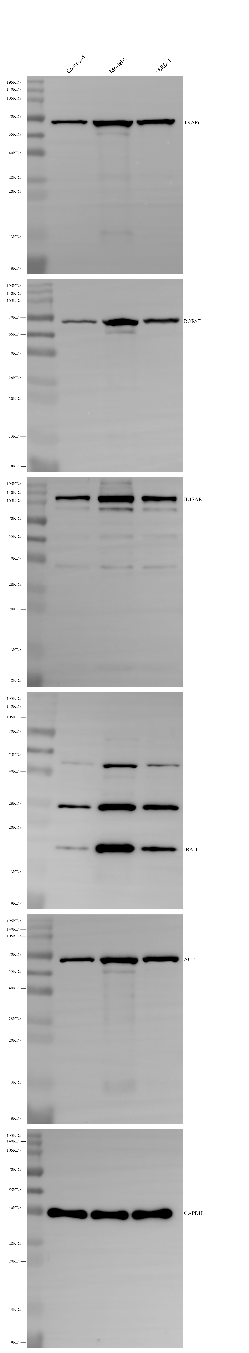

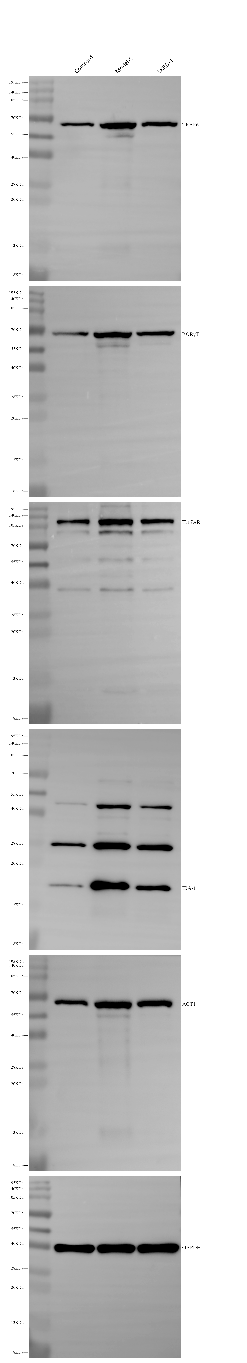


**Sample 4 Sample 5 Sample 6**

**Figure S3: Molecular docking pattern of active components to key target of microglia-mediated neuroinflammation**


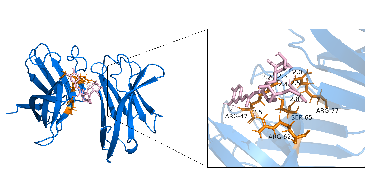

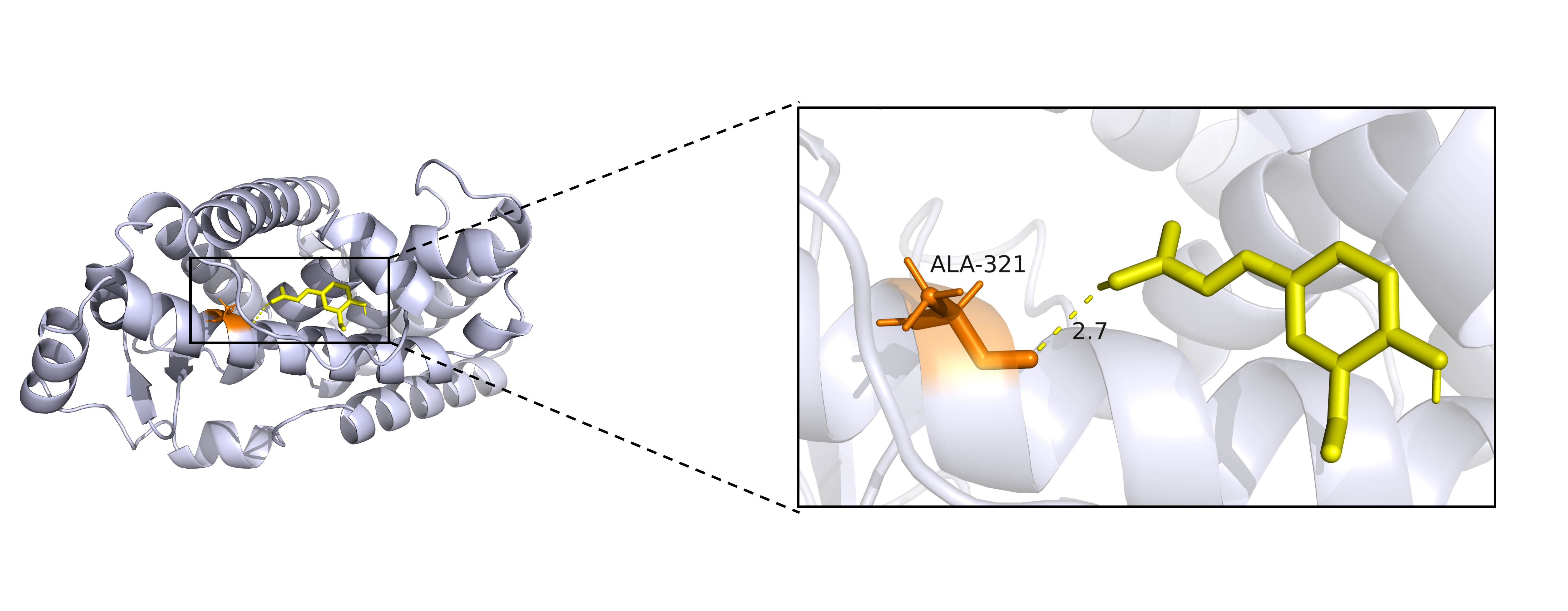

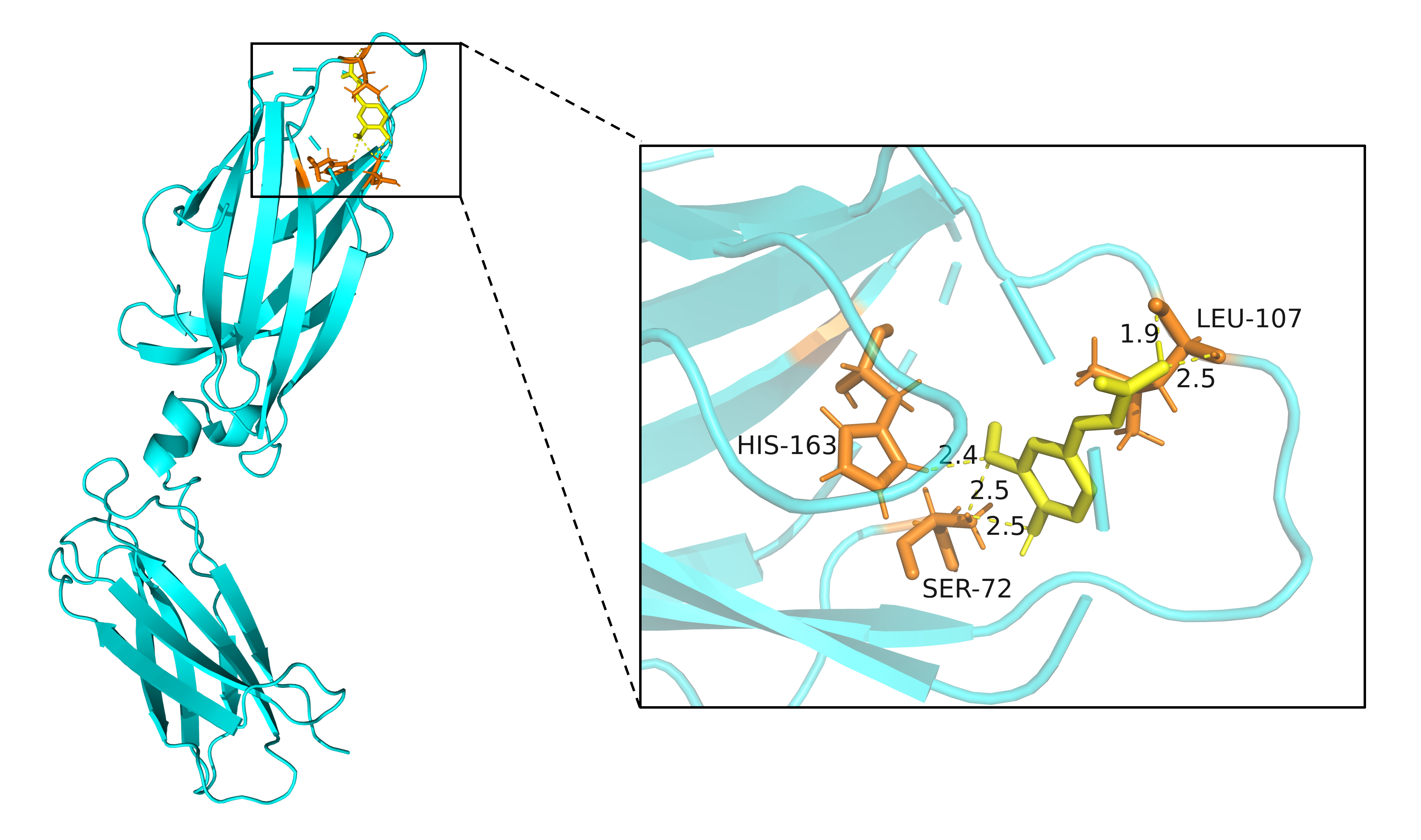

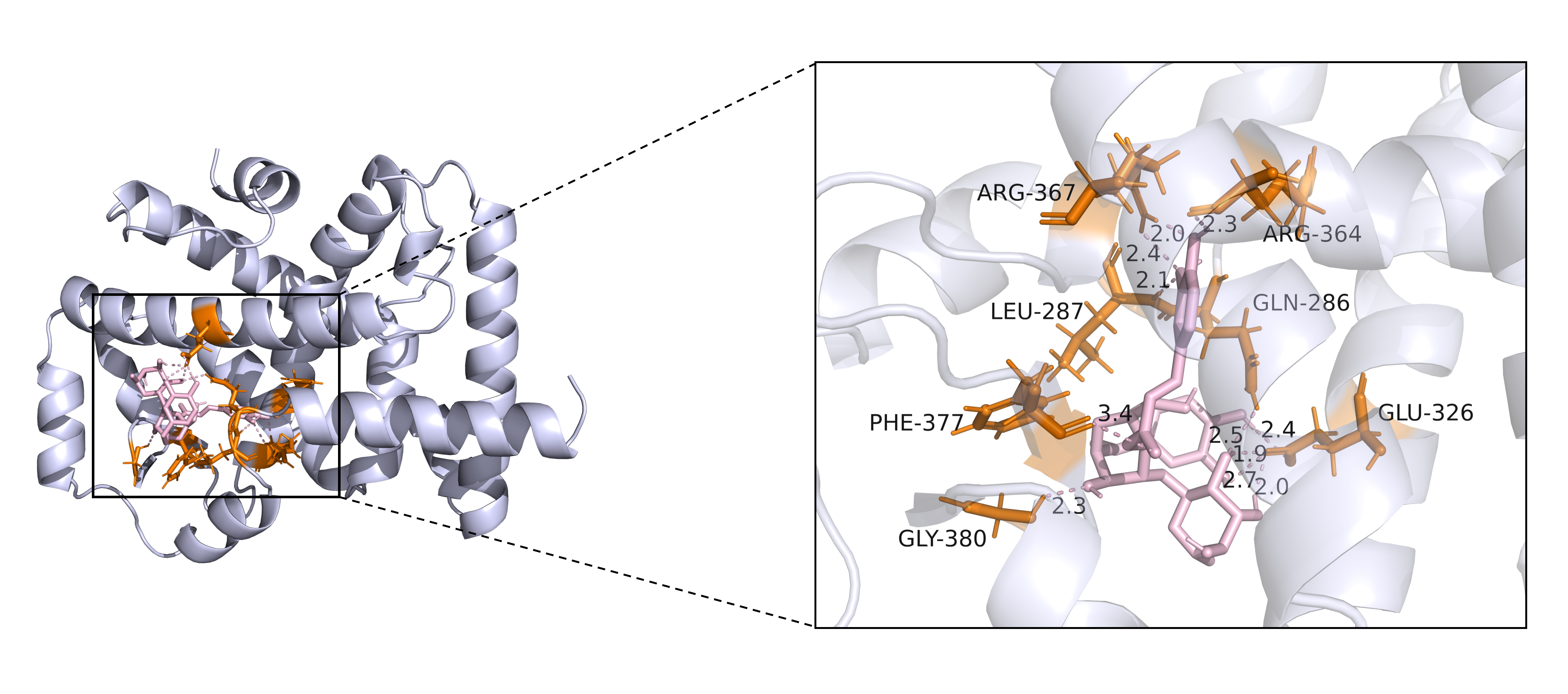


**FA**

**TREM2**

**RORγt**

**IL-17AR**


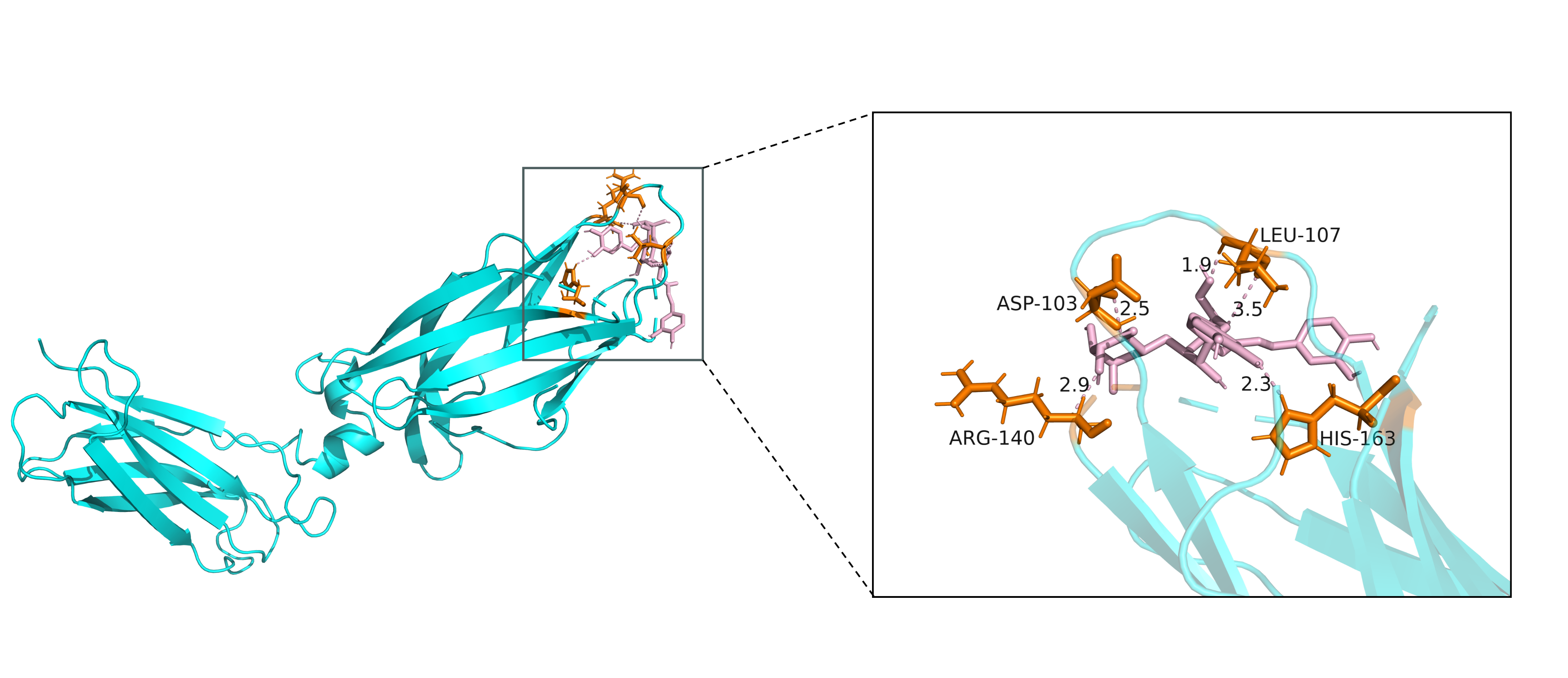

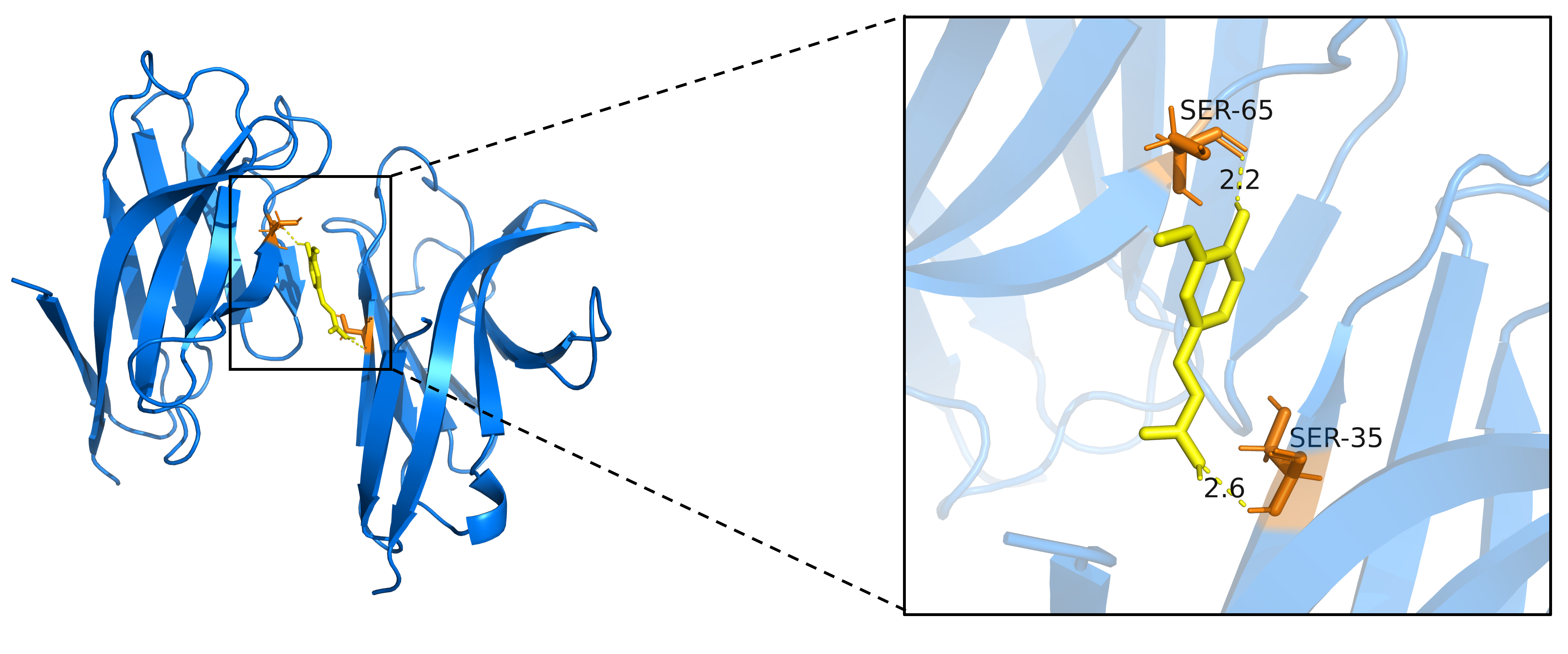


**ACT**

**Figure S4: The different dose of FA and ACT antidepressant effects on mice under stress conditions.** There were no significant differences in body weights and sucrose preference rates among the tested groups. 60 mg/kg ACT and 40 mg/kg FA were an effective dose for treatment depression. Data values were indicated as mean ± SEM (n=10 per group). ^*^*p*<0.05, ^**^*p*<0.01, ^***^*p*<0.001 vs. control group; ^#^*p*<0.05, ^##^*p*<0.01, ^###^*p*< 0.001 vs. model group.


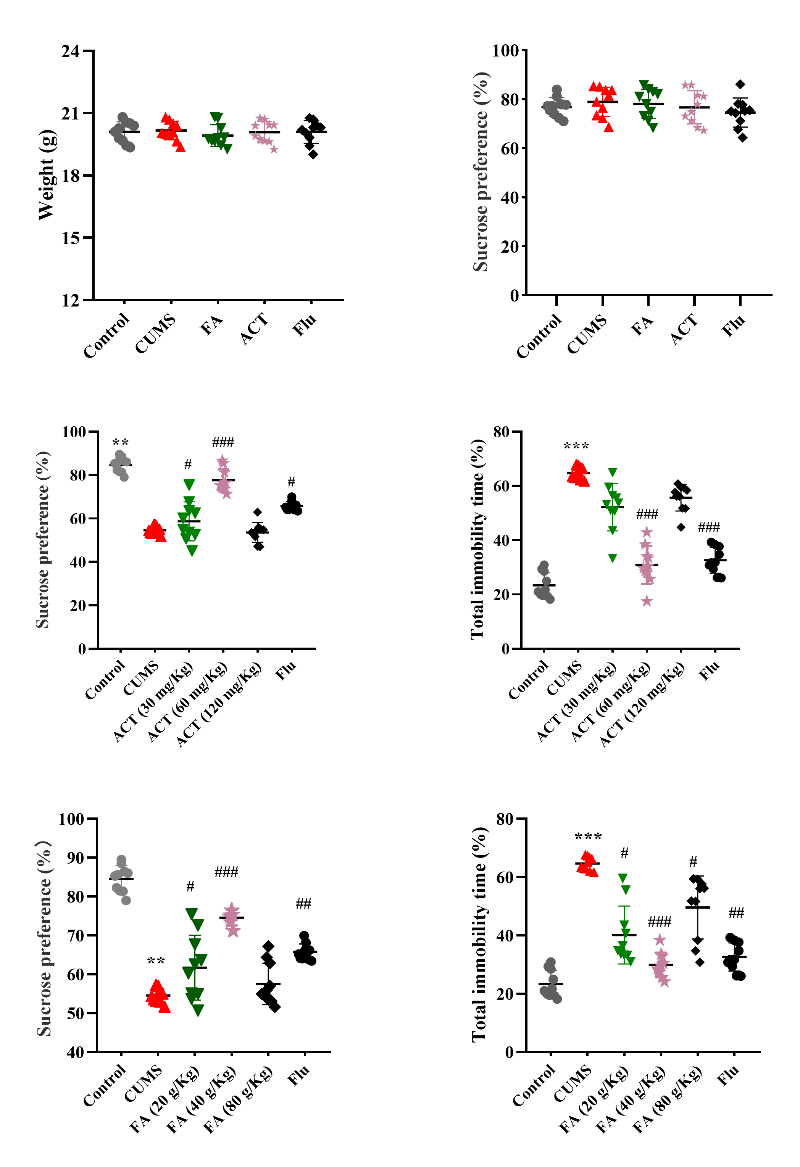


**Figure S5: Antidepressant response of ACT and FA in combination at different** **ratios.** Behavioral results indicated the antidepressant effect of ACT and FA at the equivalent dose ratio found in BDD for each compound (ACT: FA=1:9, ACT 45 mg/kg combined with FA 5 mg/kg) was superior to the other ratio compatibility. Data values were indicated as mean ± SEM (n=5 per group). ^*^*p*<0.05, ^**^*p*<0.01, ^***^*p*<0.001 vs. control group; ^#^*p*<0.05, ^##^*p*<0.01, ^###^*p*< 0.001 vs. model group.

**Figure S6:** **The pattern plot of the protein band in Figure 5C is derived from** **the whole un-cropped images of the original western blots.**

**Sample-1 Sample-2**


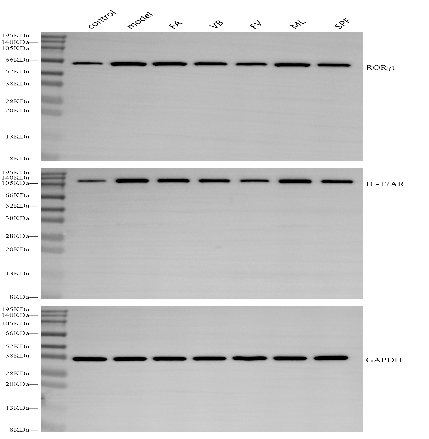

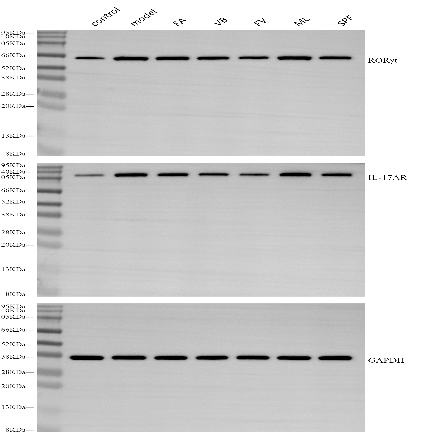


**Sample-3**  **Sample-4**


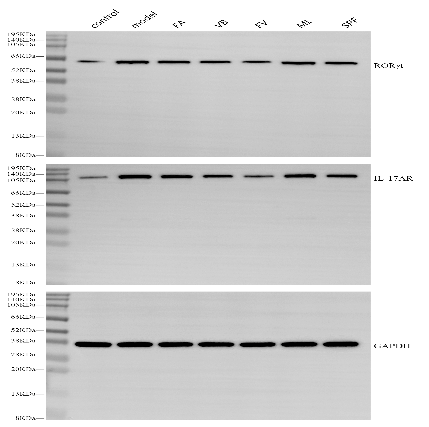

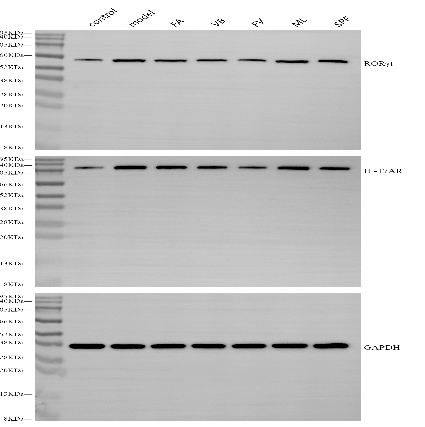


**Sample-1 Sample-2**


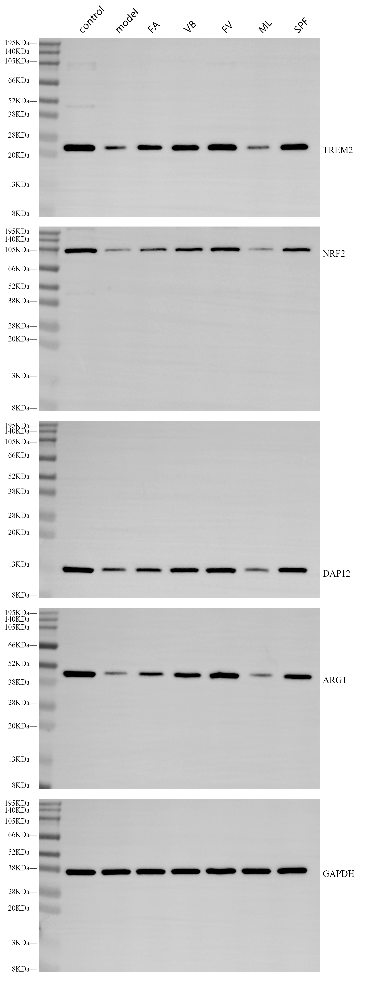

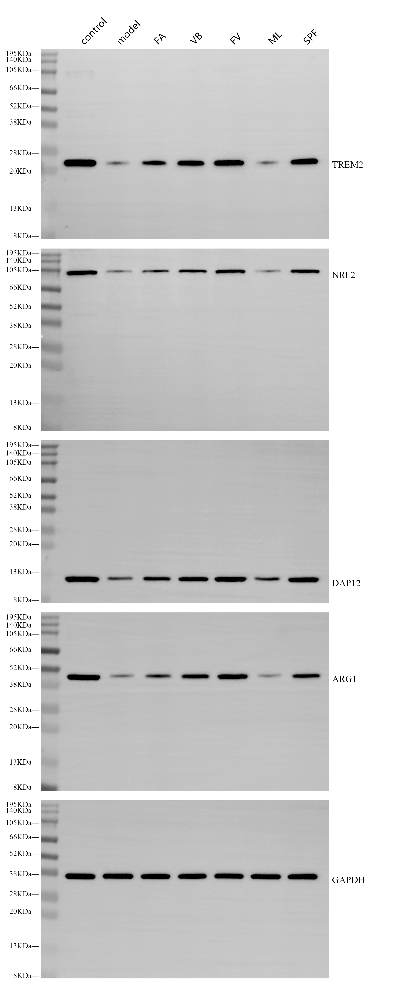


**Sample-3 Sample-4**


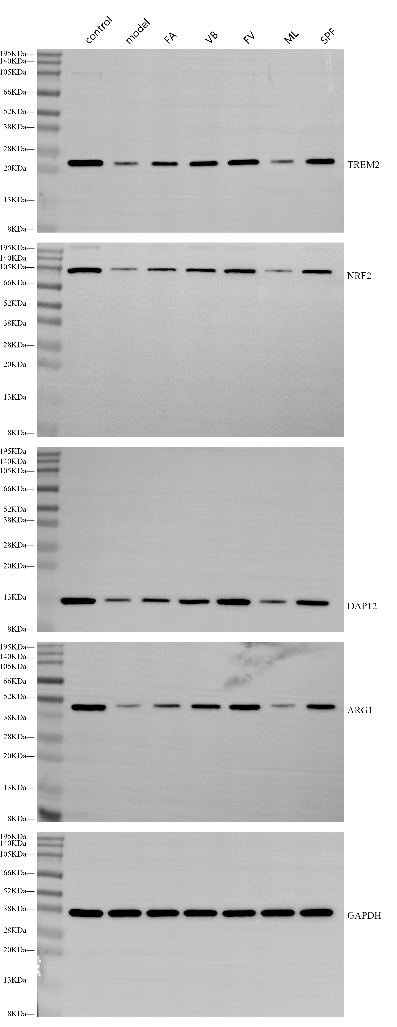

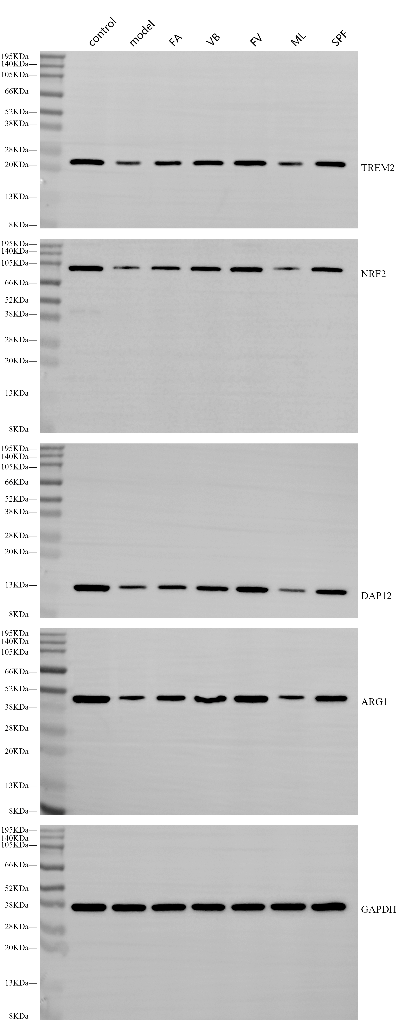


**Figure S7: ACT-FA co-formulation at original BDD ratio superiorly modulates microglial polarization and attenuates inflammatory response.**


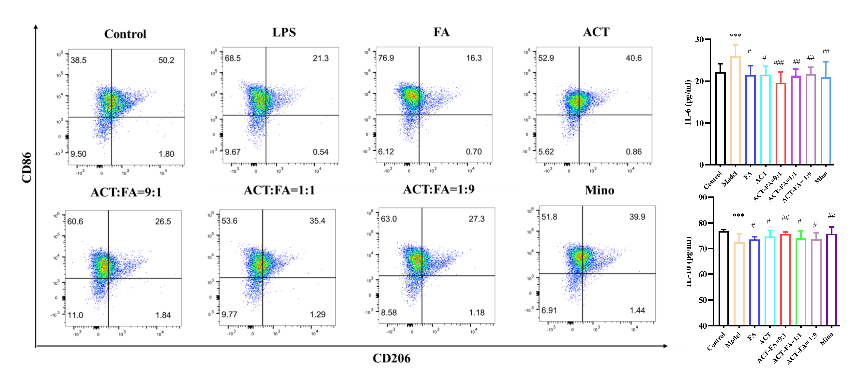


**Figure S8: The pattern plot of the protein band in Figure 7J is derived from the whole un-cropped images of the original western blots.**

**Sample-1 Sample-2**


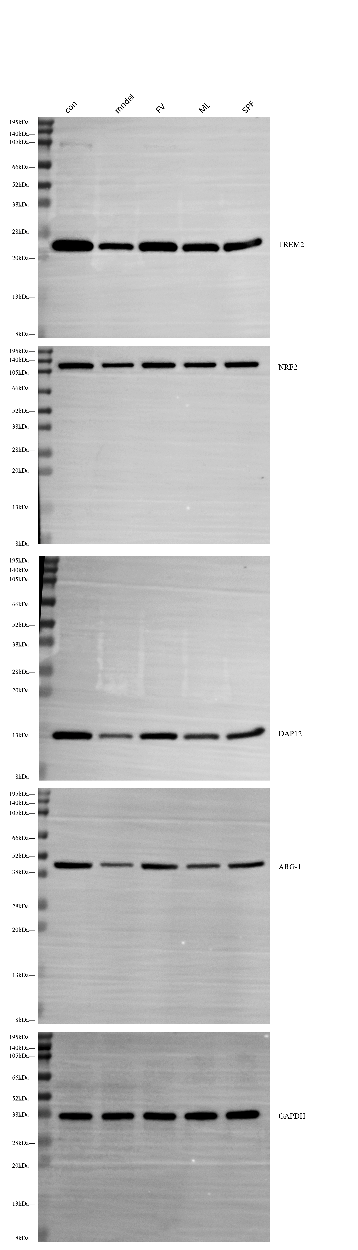

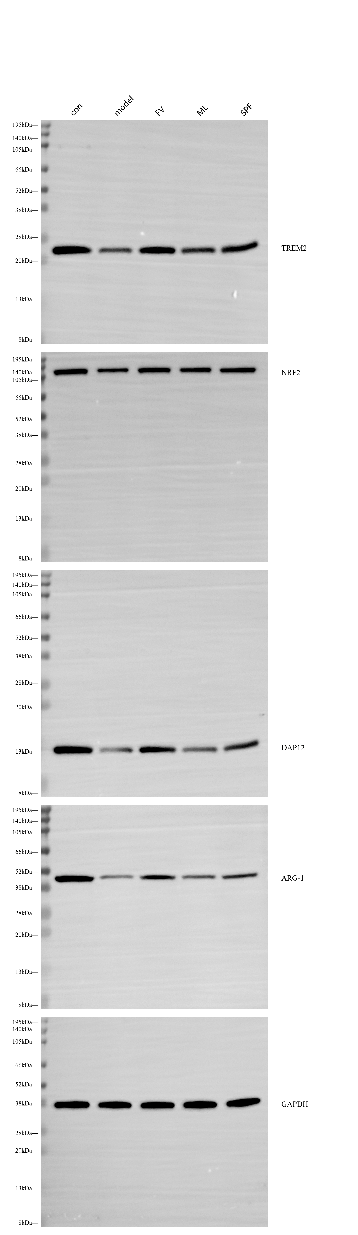


**Sample-3**


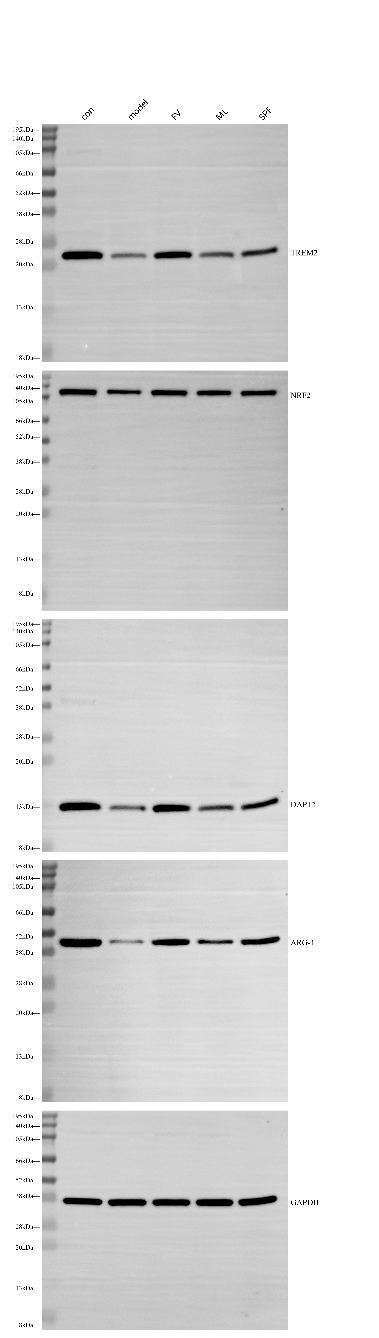


***Phytoconstituent analysis of BDD***

**Table S1. Characterization of the main chemical constituents in BDD by UPLCQTOF-MS/MS (2020.10).**

| **Number** | **Name** | **Model** | **RT [min]** | **Formula** | **m/z** |
| --- | --- | --- | --- | --- | --- |
| 1 | L-4-Hydroxyglutamate semialdehyde | + | 0.7774 | C5H9NO4 | 148.06 |
| 2 | Verbascoside | + | 0.8489 | C29H36O15 | 624.59 |
| 3 | L-2-Aminoadipic acid | + | 0.9214 | C6H11NO4 | 162.08 |
| 4 | 6-Oxopiperidine-2-carboxylic acid | + | 0.9214 | C6H9NO3 | 144.07 |
| 5 | 2,5-Furandicarboxylic acid | + | 1.315 | C6H4O5 | 139 |
| 6 | Vidarabine | + | 2.2965 | C10H13N5O4 | 268.1 |
| 7 | L-Glutamic acid | + | 1.299 | C5 H9 N O4 | 148.0602 |
| 8 | Coumarin | + | 4.8195 | C9H6O2 | 147.04 |
| 9 | 8-tridecynoic acid | + | 5.3891 | C13H22O2 | 193.16 |
| 10 | Triethyl phosphate | + | 6.2625 | C6H15O4P | 183.08 |
| 11 | Myristic acid | + | 7.5879 | C14H28O2 | 246.24 |
| 12 | (+)-15S-hydroxy-hexadecanoic acid | + | 7.6919 | C16H32O3 | 290.27 |
| 13 | 17-hydroxy stearic acid | + | 9.3046 | C18H36O3 | 318.3 |
| 14 | 14:0(5Me[R],9Me[R],13Me) | + | 9.6715 | C17H34O2 | 288.29 |
| 15 | Stearic acid | + | 10.2174 | C18H36O2 | 302.31 |
| 16 | 2E,6Z,8Z,12E-hexadecatetraenoc acid | + | 11.2287 | C16H24O2 | 249.18 |
| 17 | Monoethylhexyl phthalic acid | + | 11.6635 | C16H22O4 | 301.14 |
| 18 | Polidocanol | + | 12.2376 | C30H62O10 | 600.47 |
| 19 | 1-O-(2R-hydroxy-hexadecyl)-sn-glycerol | + | 12.9221 | C19H40O4 | 333.3 |
| 20 | Oleamide | + | 13.1112 | C18H35NO | 304.26 |
| 21 | Palmitic acid | - | 22.78 | C16 H32 O2 | 256.2402 |
| 22 | Azelaic acid | - | 13.518 | C9 H16 O4 | 187.09688 |
| 23 | Kojibiose | - | 0.8957 | C5H9NO4 | 341.11 |
| 24 | Maltotriose | - | 0.9073 | C18H32O16 | 549.17 |
| 25 | Maltotetraose | - | 1.0472 | C24H42O21 | 711.22 |
| 26 | Osmundalactone | - | 1.1405 | C6H8O3 | 173.05 |
| 27 | Ferulic acid | - | 11.898 | C10 H10 O4 | 193.04987 |
| 28 | Citric acid | - | 2.814 | C6 H8 O7 | 132.005 |
| 29 | Catalpol | - | 1.9963 | C15H22O10 | 407.12 |
| 30 | Ornithine | - | 1.187 | C5 H12 N2 O2 | 131.08134 |
| 31 | Dihydroartemisinin | - | 4.9027 | C15H24O5 | 283.15 |
| 32 | Caffeic acid | - | 10.265 | C9 H8 O4 | 180.0414 |

**2021.10**

| **Number** | **Name** | **Model** | **RT [min]** | **Formula** | **m/z** |
| --- | --- | --- | --- | --- | --- |
| 1 | N-Ethylglycine | - | 1.305 | C4 H9 N O2 | 102.05454 |
| 2 | D-Glucose 6-phosphate | - | 1.715 | C6 H13 O9 P | 259.02213 |
| 3 | D-(+)-Malic acid | - | 1.726 | C4 H6 O5 | 133.01279 |
| 4 | 4-Oxoproline | - | 2.713 | C5 H7 N O3 | 128.03395 |
| 5 | Citric acid | - | 2.713 | C6 H8 O7 | 191.01866 |
| 6 | 2-Furoic acid | - | 2.713 | C5 H4 O3 | 111.0073 |
| 7 | Catalpol | - | 1.9963 | C15H22O10 | 407.12 |
| 8 | Geniposidic acid | - | 7.833 | C16 H22 O10 | 419.11911 |
| 9 | Bioside | - | 8.493 | C20 H30 O12 | 461.16595 |
| 10 | Ornithine | - | 1.187 | C5 H12 N2 O2 | 131.08134 |
| 11 | 2-(Acetylamino)hexanoic acid | - | 10.551 | C8 H15 N O3 | 172.09689 |
| 12 | 6-O-Feruloylcatalpol | - | 10.738 | C25 H30 O13 | 537.16107 |
| 13 | Ferulic acid | - | 11.874 | C10 H10 O4 | 193.04971 |
| 14 | verbascoside | - | 11.925 | C29 H36 O15 | 623.19788 |
| 15 | Phenylacetaldehyde | - | 12.541 | C8 H8 O | 119.04877 |
| 16 | Azelaic acid | - | 13.461 | C9 H16 O4 | 187.09676 |
| 17 | DEET | - | 15.232 | C12 H17 N O | 192.13809 |
| 18 | (±)9-HpODE | - | 18.279 | C18 H32 O4 | 311.22266 |
| 19 | Caffeic acid | - | 10.265 | C9 H8 O4 | 180.0414 |
| 20 | 2,2'-Methylenebis(4-methyl-6-tert-butylphenol) | - | 21.261 | C23 H32 O2 | 339.23248 |
| 21 | Corticosterone | - | 22.489 | C21 H30 O4 | 391.21539 |
| 22 | Palmitic acid | - | 22.78 | C16 H32 O2 | 256.2402 |
| 23 | Stearic acid | - | 24.069 | C18 H36 O2 | 283.26419 |
| 24 | L-Histidine | + | 1.159 | C6 H9 N3 O2 | 156.07671 |
| 25 | DL-Arginine | + | 1.165 | C6 H14 N4 O2 | 175.11879 |
| 26 | Choline | + | 1.175 | C5 H13 N O | 104.10734 |
| 27 | DL-Glutamine | + | 1.277 | C5 H10 N2 O3 | 147.07631 |
| 28 | L-Glutamic acid | + | 1.299 | C5 H9 N O4 | 148.0602 |
| 29 | D-(+)-Proline | + | 1.392 | C5 H9 N O2 | 116.07081 |
| 30 | Trigonelline | + | 1.45 | C7 H7 N O2 | 138.05486 |
| 31 | Maltotriose | + | 1.538 | C18 H32 O16 | 505.17587 |
| 32 | L-Iditol | + | 1.552 | C6 H14 O6 | 183.08623 |
| 33 | L-2-Aminoadipic acid | + | 1.632 | C6 H11 N O4 | 162.07584 |
| 34 | Nicotinamide | + | 2.134 | C6 H6 N2 O | 123.05556 |
| 35 | L-Isoleucine | + | 2.514 | C6 H13 N O2 | 132.10184 |
| 36 | D-(+)-Pyroglutamic Acid | + | 2.514 | C5 H7 N O3 | 130.0499 |
| 37 | Pyridoxine | + | 4.341 | C8 H11 N O3 | 170.08105 |
| 38 | Adenosine | + | 4.603 | C10 H13 N5 O4 | 268.1037 |
| 39 | L-Phenylalanine | + | 5.317 | C9 H11 N O2 | 166.08615 |
| 40 | 2,4-Xylidine | + | 6.581 | C8 H11 N | 122.0965 |
| 41 | 4-Indolecarbaldehyde | + | 7.321 | C9 H7 N O | 146.05988 |
| 42 | D-(+)-Tryptophan | + | 7.321 | C11 H12 N2 O2 | 205.09685 |
| 43 | Caprolactam | + | 8.417 | C6 H11 N O | 114.09148 |
| 44 | 2-(4-Hydroxyphenyl)ethyl 6-O-[(2R,3R,4R)-3,4-dihydroxy-4-(hydroxymethyl)tetrahydro-2-furanyl]-beta-D-glucopyranoside | + | 9.268 | C19 H28 O11 | 450.1973 |
| 45 | Triethyl phosphate | + | 12.729 | C6 H15 O4 P | 183.07802 |
| 46 | Dimethyl phthalate | + | 13.368 | C10 H10 O4 | 195.06509 |
| 47 | 3-ethyl-4-hydroxy-1-methyl-1,2-dihydroquinolin-2-one | + | 13.849 | C12 H13 N O2 | 204.10181 |
| 48 | (2E)-3-(2-{[(2S,3R,4S,5S,6R)-3,4,5-trihydroxy-6-(hydroxymethyl)oxan-2-yl]oxy}phenyl)prop-2-enoic acid | + | 14.859 | C15 H18 O8 | 309.09634 |
| 49 | Monobutyl phthalate | + | 15.469 | C12 H14 O4 | 223.09631 |
| 50 | (3S,4aR,5R,6R)-3,6-dihydroxy-4a,5-dimethyl-3-(prop-1-en-2-yl)-2,3,4,4a,5,6,7,8-octahydronaphthalen-2-one | + | 15.978 | C15 H22 O3 | 233.15329 |
| 51 | Dipropyleneglycol dibenzoate | + | 18.277 | C20 H22 O5 | 365.13544 |
| 52 | Diisobutylphthalate | + | 18.63 | C16 H22 O4 | 279.15857 |
| 53 | 1-Dodecyl-2-pyrrolidinone | + | 21.201 | C16 H31 N O | 254.24728 |
| 54 | Stearamide | + | 22.701 | C18 H37 N O | 284.29434 |
| 55 | 1-Stearoylglycerol | + | 23.136 | C21 H42 O4 | 381.29654 |
| 56 | Diisooctyl phthalate | + | 23.153 | C24 H38 O4 | 391.28333 |

**2022.10**

| **Number** | **Name** | **Model** | **RT [min]** | **Formula** | **m/z** |
| --- | --- | --- | --- | --- | --- |
| 1 | Ornithine | - | 1.18 | C5 H12 N2 O2 | 132.0887 |
| 2 | L-Histidine | - | 1.265 | C6 H9 N3 O2 | 155.0684 |
| 3 | D-(-)-Mannitol | - | 1.324 | C6 H14 O6 | 181.0708 |
| 4 | L-Glutamic acid | - | 1.403 | C5 H9 N O4 | 146.0447 |
| 5 | Gluconic acid | - | 1.412 | C6 H12 O7 | 136.0364 |
| 6 | Isocitric acid | - | 1.675 | C6 H8 O7 | 192.0262 |
| 7 | D-(+)-Malic acid | - | 1.772 | C4 H6 O5 | 134.0202 |
| 8 | D-Glucose 6-phosphate | - | 1.813 | C6 H13 O9 P | 260.0297 |
| 9 | 4-Oxoproline | - | 2.805 | C5 H7 N O3 | 129.0414 |
| 10 | Citric acid | - | 2.814 | C6 H8 O7 | 132.005 |
| 11 | 2-Furoic acid | - | 2.815 | C5 H4 O3 | 130.0253 |
| 12 | 1,2,3-cyclopropanetricarboxylic acid | - | 5.198 | C6 H6 O6 | 174.0155 |
| 13 | Guanosine | - | 5.402 | C10 H13 N5 O5 | 283.0919 |
| 14 | Methyl acetoacetate | - | 5.49 | C5 H8 O3 | 116.046 |
| 15 | Citraconic acid | - | 5.987 | C5 H6 O4 | 130.0253 |
| 16 | Methylsuccinic acid | - | 6.336 | C5 H8 O4 | 132.041 |
| 17 | Catalpol | - | 1.9963 | C15H22O10 | 407.12 |
| 18 | Geniposidic acid | - | 7.847 | C16 H22 O10 | 374.1212 |
| 19 | Bioside | - | 8.308 | C20 H30 O12 | 462.174 |
| 20 | 4-Acetyl-3-hydroxy-5-methylphenyl β-D-glucopyranoside | - | 9.053 | C15 H20 O8 | 328.1162 |
| 21 | Isophthalic acid | - | 10.137 | C8 H6 O4 | 166.0255 |
| 22 | Caffeic acid | - | 10.265 | C9 H8 O4 | 180.0414 |
| 23 | Phlinoside A | - | 10.618 | C35 H46 O20 | 785.2515 |
| 24 | Perillic acid | - | 11.016 | C10 H14 O2 | 166.098 |
| 25 | 2-(4-Hydroxyphenyl)ethyl 6-O-[(2R,3R,4R)-3,4-dihydroxy-4-(hydroxymethyl)tetrahydro-2-furanyl]-beta-D-glucopyranoside | - | 11.074 | C19 H28 O11 | 478.1686 |
| 26 | Methyl 1-(hexopyranosyloxy)-4a-hydroxy-7-methyl-5-oxo-1,4a,5,6,7,7a-hexahydrocyclopenta[c]pyran-4-carboxylate | - | 11.222 | C17 H24 O11 | 404.1315 |
| 27 | Phenylacetaldehyde | - | 11.558 | C8 H8 O | 120.0563 |
| 28 | Verbascoside |  | 11.954 | C29 H36 O15 | 624.2055 |
| 29 | 2,3-Dihydro-1-benzofuran-2-carboxylic acid | - | 12.581 | C9 H8 O3 | 118.0407 |
| 30 | Ferulic acid | - | 12.736 | C10 H10 O4 | 134.036 |
| 31 | 1-Hydroxybenzotriazol | - | 12.738 | C6 H5 N3 O | 135.0433 |
| 32 | Azelaic acid | - | 13.515 | C9 H16 O4 | 188.1042 |
| 33 | 4-Indolecarbaldehyde | - | 15.543 | C9 H7 N O | 145.0516 |
| 34 | 2-(4-Methyl-3-cyclohexen-1-yl)-2-propanyl 6-O-(6-deoxy-α-L-mannopyranosyl)-β-D-glucopyranoside | - | 15.62 | C22 H38 O10 | 508.2521 |
| 35 | 4,4-Diphenylmethane diisocyanate | - | 15.929 | C15 H10 N2 O2 | 250.074 |
| 36 | (15Z)-9,12,13-Trihydroxy-15-octadecenoic acid | - | 16.478 | C18 H34 O5 | 330.2407 |
| 37 | 5-[(3Z)-5-Hydroxy-3-methyl-3-penten-1-yl]-1,4a-dimethyl-6-methylenedecahydro-1-naphthalenecarboxylic acid | - | 18.496 | C20 H32 O3 | 320.2352 |
| 38 | (+/-)9,10-dihydroxy-12Z-octadecenoic acid | - | 19.558 | C18 H34 O4 | 295.228 |
| 39 | Linoleic Acid | - | 22.284 | C18 H32 O2 | 280.2403 |
| 40 | Palmitic acid | - | 22.78 | C16 H32 O2 | 256.2402 |
| 41 | Oleic acid | - | 23.044 | C18 H34 O2 | 281.2487 |
| 42 | DL-Arginine | + | 1.391 | C6 H14 N4 O2 | 175.1189 |
| 43 | Choline | + | 1.403 | C5 H13 N O | 104.1075 |
| 44 | D-(+)-Proline | + | 1.431 | C5 H9 N O2 | 116.0709 |
| 45 | DL-Glutamine | + | 1.439 | C5 H10 N2 O3 | 147.0764 |
| 46 | Methyl isonicotinate | + | 1.497 | C7 H7 N O2 | 138.055 |
| 47 | L-2-Aminoadipic acid | + | 1.589 | C6 H11 N O4 | 162.076 |
| 48 | Nicotinamide | + | 2.217 | C6 H6 N2 O | 123.0556 |
| 49 | Adenosine 5'-monophosphate | + | 2.566 | C10 H14 N5 O7 P | 348.0702 |
| 50 | D-(+)-Pyroglutamic Acid | + | 2.783 | C5 H7 N O3 | 130.05 |
| 51 | 4-Hydroxybenzaldehyde | + | 2.964 | C7 H6 O2 | 123.0444 |
| 52 | Adenosine | + | 4.688 | C10 H13 N5 O4 | 268.1038 |
| 53 | Adenosine 3'5'-cyclic monophosphate | + | 5.174 | C10 H12 N5 O6 P | 330.0596 |
| 54 | L-Phenylalanine | + | 5.388 | C9 H11 N O2 | 166.0862 |
| 55 | Pyridoxal | + | 6.97 | C8 H9 N O3 | 168.0654 |
| 56 | DL-Tryptophan | + | 7.367 | C11 H12 N2 O2 | 188.0704 |
| 57 | Adenine | + | 8.068 | C5 H5 N5 | 136.0618 |
| 58 | Mussaenosidic acid | + | 8.823 | C16 H24 O10 | 375.1297 |
| 59 | Benzyl 6-O-beta-D-glucopyranosyl-beta-D-glucopyranoside | + | 9.37 | C19 H28 O11 | 477.162 |
| 60 | (2E)-3-(2-{[(2S,3R,4S,5S,6R)-3,4,5-trihydroxy-6-(hydroxymethyl)oxan-2-yl]oxy}phenyl)prop-2-enoic acid | + | 10.989 | C15 H18 O8 | 309.0963 |
| 61 | 4-Methylumbelliferyl-α-D-glucopyranoside | + | 11.245 | C16 H18 O8 | 339.1067 |
| 62 | (1R,9S)-11-(Cyclohexylcarbonyl)-5-(2-methylphenyl)-7,11-diazatricyclo[7.3.1.02,7]trideca-2,4-dien-6-one | + | 12.727 | C25 H30 N2 O2 | 413.2138 |
| 63 | 2-Hydroxycinnamic acid | + | 14.889 | C9 H8 O3 | 147.044 |
| 64 | Bis(4-ethylbenzylidene) sorbitol | + | 17.249 | C24 H30 O6 | 415.2109 |
| 65 | Prostaglandin F2α 1-11-lactone | + | 19.24 | C20 H32 O4 | 317.2121 |
| 66 | 18-β-Glycyrrhetinic acid | + | 19.835 | C30 H46 O4 | 469.3326 |
| 67 | Oleic acid alkyne | + | 21.614 | C18 H30 O2 | 277.2175 |

**2023.10**

| **Number** | **Name** | **Model** | **RT [min]** | **Formula** | **m/z** |
| --- | --- | --- | --- | --- | --- |
| 1 | Ornithine | - | 1.187 | C5 H12 N2 O2 | 131.08134 |
| 2 | L-Histidine | - | 1.297 | C6 H9 N3 O2 | 154.06107 |
| 3 | D-(+)-Pyroglutamic Acid | - | 1.444 | C5 H7 N O3 | 147.07635 |
| 4 | Trigonelline | - | 1.459 | C7 H7 N O2 | 138.05492 |
| 5 | 4-Oxoproline | - | 2.676 | C5 H7 N O3 | 128.03406 |
| 6 | Methyl acetoacetate | - | 5.4 | C5 H8 O3 | 115.03872 |
| 7 | L-Glutamic acid | - | 1.403 | C5 H9 N O4 | 146.0447 |
| 8 | Benzyl 6-O-beta-D-glucopyranosyl-beta-D-glucopyranoside | - | 9.364 | C19 H28 O11 | 477.16165 |
| 9 | Caffeic acid | - | 10.273 | C9 H8 O4 | 179.03406 |
| 10 | Catalpol | - | 1.9963 | C15H22O10 | 407.12 |
| 11 | Ferulic acid | - | 11.898 | C10 H10 O4 | 193.04987 |
| 12 | Phenylacetaldehyde | - | 12.581 | C8 H8 O | 119.04887 |
| 13 | 2,3-Dihydro-1-benzofuran-2-carboxylic acid | - | 12.582 | C9 H8 O3 | 163.03891 |
| 14 | 1-Hydroxybenzotriazol | - | 12.737 | C6 H5 N3 O | 134.03606 |
| 15 | Azelaic acid | - | 13.518 | C9 H16 O4 | 187.09688 |
| 16 | Citric acid | - | 2.814 | C6 H8 O7 | 132.005 |
| 17 | Prostaglandin F2α 1-11-lactone | - | 19.249 | C20 H32 O4 | 317.21234 |
| 18 | Taurochenodeoxycholic Acid (sodium salt) | - | 19.881 | C26 H45 N O6 S | 498.28989 |
| 19 | Stearic acid | - | 24.164 | C18 H36 O2 | 283.2644 |
| 20 | Choline | + | 1.38 | C5 H13 N O | 104.10742 |
| 21 | Valine | + | 1.592 | C5 H11 N O2 | 118.08643 |
| 22 | L-Glutathione oxidized | + | 3.909 | C20 H32 N6 O12 S2 | 307.08322 |
| 23 | Adenosine | + | 4.689 | C10 H13 N5 O4 | 268.10382 |
| 24 | L-Phenylalanine | + | 5.391 | C9 H11 N O2 | 166.08614 |
| 25 | DL-Tryptophan | + | 7.373 | C11 H12 N2 O2 | 188.0704 |
| 26 | Adenine | + | 8.067 | C5 H5 N5 | 136.06165 |
| 27 | Ethyl levulinate | + | 8.27 | C7 H12 O3 | 145.08597 |
| 28 | L-2-Aminoadipic acid | + | 1.589 | C6 H11 N O4 | 162.076 |
| 29 | Verbascoside | + | 0.8489 | C29H36O15 | 624.59 |
| 30 | 4-Coumaric acid | + | 12.571 | C9 H8 O3 | 165.05455 |
| 31 | 4-Methylumbelliferyl-α-D-glucopyranoside | + | 12.846 | C16 H18 O8 | 339.10672 |
| 32 | Palmitic Acid | + | 14.813 | C16 H32 O2 | 274.27362 |
| 33 | Cuminaldehyde | + | 17.248 | C10 H12 O | 149.0959 |
| 34 | Diisobutylphthalate | + | 18.676 | C16 H22 O4 | 301.14056 |
| 35 | 4-Indolecarbaldehyde | + | 10.885 | C9 H7 N O | 146.0598 |

**Table S2： DEGs and their characteristics of mPFC-NAc and colon tissues from mice under CUMS after LBRD administration.**

| Symbol | Gene ID | Length (bp) | Chromosomal Map | Fold Change LBRD/CUMS | *P*-value | Description |  |
| --- | --- | --- | --- | --- | --- | --- | --- |
| mPFC |  |  |  |  |  |  |  |
| Calml4 | 75600 | 18067 | 9 B | 3.710 | 0.002 | calmodulin-like 4 |  |
| Btg2 | 12227 | 4290 | 1 E4 | 3.176 | 0.007 | B cell translocation gene 2 |  |
| Ntrk2 | 18212 | 323380 | 13 B1 | 2.821 | 0.042 | neurotrophic receptor tyrosine kinase 2 |  |
| Arc | 11838 | 3489 | 15 D3 | 2.790 | 0.013 | activity regulated cytoskeletal-associated protein |  |
| Npas4 | 225872 | 17576 | 19 A | 2.766 | 0.006 | neuronal PAS domain protein 4 |  |
| Mbp | 17196 | 110514 | 2 H1 | 2.342 | 0.002 | myelin basic protein |  |
| Slc6a11 | 243616 | 118645 | 6 E3 | 2.294 | 0.005 | neurotransmitter transporter, GABA |  |
| Slc32a1 | 22348 | 4989 | 2 H1 | 2.243 | 0.002 | GABA vesicular transporter |  |
| Mtg2 | 52856 | 15309 | 2 H4 | 2.091 | 0.007 | mitochondrial ribosome associated GTPase 2 |  |
| Cldn5 | 12741 | 1416 | 16 A3 | 1.981 | 0.004 | Claudin 5 |  |
| Vglut1 | 72961 | 12218 | 7 B3 | 1.825 | 0.003 | vesicular glutamate transporters |  |
| Ntf3 | 18205 | 65379 | 6 F3 | 1.760 | 0.013 | neurotrophin 3 |  |
| Bdnf | 12064 | 52343 | 2 E3 | 1.734 | 0.038 | brain derived neurotrophic factor |  |
| Fabp7 | 12140 | 3527 | 10 B4 | 1.593 | 0.013 | fatty acid binding protein 7, brain |  |
| Gad1 | 14415 | 40249 | 2 C2 | 1.574 | 0.004 | glutamate decarboxylase 1 |  |
| PSD95 | 13385 | 26927 | 11 B3 | 1.561 | 0.002 | discs large MAGUK scaffold protein 4 |  |
| Nr1d1 | 217166 | 7445 | 11 D | 0.686 | 0.007 | nuclear receptor subfamily |  |
| Hbb-b1 | 15129 | 1399 | 7 E3 | 0.674 | 0.015 | hemoglobin, beta |  |
| Hba-a1 | 15122 | 814 | 11 A4 | 0.667 | 0.004 | hemoglobin alpha, adult chain 1 |  |
| Irf7 | 54123 | 3316 | 7 F5 | 0.627 | 0.003 | interferon regulatory factor 7 |  |
| Dbp | 13170 | 5110 | 7 B3 | 0.625 | 0.015 | D site albumin promoter binding protein |  |
| Acvr1c | 269275 | 90445 | 2 C1 | 0.497 | 0.005 | activin A receptor, type IC |  |
| Flot2 | 14252 | 22593 | 11 B5 | 0.487 | 0.007 | flotillin 2 |  |
| Syt6 | 54524 | 70325 | 3 F2 | 0.376 | 0.003 | synaptotagmin VI |  |
| Il1b | 16176 | 6570 | 2 F1 | 0.459 | 0.006 | Interleukin 1 beta |  |
| NAc |  |  |  |  |  |  |  |
| Avp | 11998 | 1969 | 2 F1 | 7.51 | 0.014 | Arginine vasopressin |  |
| Sigmar1 | 18391 | 2867 | 4 A5 | 1.83 | 0.009 | Sigma non-opioid intracellular receptor 1 |  |
| Lcn2 | 16819 | 3103 | 2 B | 1.66 | 0.023 | Lipocalin 2 |  |
| Ccl17 | 20295 | 2671 | 8 C5 | 1.62 | 0.038 | Chemokine (C-C motif) ligand 17 |  |
| Tnnc1 | 21924 | 3418 | 14 B | 1.61 | 0.011 | Troponin C, cardiac/slow skeletal |  |
| Gphn | 268566 | 467448 | 12 C3 | 1.61 | 0.035 | Gephyrin |  |
| Cldn5 | 12741 | 1416 | 16 A3 | 1.58 | 0.008 | Claudin 5 |  |
| Etnk2 | 214253 | 16856 | 1 E4 | 1.53 | 0.003 | Ethanolamine kinase 2 |  |
| Sphk1 | 20698 | 5841 | 11 E2 | 1.52 | 0.034 | Sphingosine kinase 1 |  |
| Slc32a1 | 22348 | 8488 | 2 H1 | 1.51 | 0.009 | GABA vesicular transporter |  |
| Foxo1 | 56458 | 81773 | 3 C | 1.5 | 0.011 | Forkhead box O1 |  |
| Nmbr | 18101 | 12309 | 10 A2 | 0.64 | 0.046 | Neuromedin B receptor |  |
| Bdnf | 12064 | 52344 | 2 E3 | 0.64 | 0.029 | Brain derived neurotrophic factor |  |
| Cacng1 | 12299 | 13259 | 11 E1 | 0.63 | 0.016 | Calcium channel, voltage-dependent, gamma subunit 1 |  |
| Mboat1 | 218121 | 110383 | 13 A3 | 0.62 | 0.003 | Membrane bound O-acyltransferase domain containing 1 |  |
| Ccl3 | 20302 | 1536 | 11 C | 0.625 | 0.023 | Chemokine (C-C motif) ligand 3 |  |
| Il1b | | 16176 | 6570 | 2 F1 | 0.62 | 0.002 | Interleukin 1 beta |
| Ak7 | 78801 | 78592 | 12 E | 0.61 | 0.003 | Adenylate kinase 7 |  |
| Ncam1 | 17967 | 297481 | 9A 5.3 | 0.59 | 0.007 | Neural cell adhesion molecule 1 |  |
| Lbp | 16803 | 26360 | 2 H1 | 0.59 | 0.024 | Lipopolysaccharide binding protein |  |
| Glp1r | 14652 | 34644 | 17 A3.3 | 0.59 | 0.007 | Glucagon-like peptide 1 receptor |  |
| Arc | 11838 | 3490 | 15 D3 | 0.58 | 0.012 | Activity regulated cytoskeletal-associated protein |  |
| Creb5 | 231991 | 413196 | 6 B3 | 0.57 | 0.029 | cAMP responsive element binding protein 5 |  |
| Fos | 14281 | 3385 | 12 D2 | 0.57 | 0.014 | FBJ osteosarcoma oncogene |  |
| Hdac1 | 433759 | 26543 | 4 D2.2 | 0.55 | 0.042 | Histone deacetylase 1 |  |
| Col6a3 | 12835 | 77129 | 1 D | 0.54 | 0.042 | Collagen, type VI, alpha 3 |  |
| Shisa6 | 380702 | 316165 | 11 B3 | 0.47 | 0.047 | Shisa family member 6 |  |
| Thbs4 | 21828 | 43233 | 13 C3 | 0.44 | 0.041 | Thrombospondin 4 |  |
| H4c9 | 319158 | 403 | 13 A3.1 | 0.43 | 0.006 | H4 clustered histone 9 |  |
| Calml4 | 75600 | 18136 | 9 B | 0.38 | 0.031 | Calmodulin-like 4 |  |
| Drd3 | 13490 | 72069 | 16 B4 | 0.19 | 0.015 | Dopamine receptor D3 |  |
| Colon |  |  |  |  |  |  |  |
| Clps | 109791 | 2557 | 17 A 3.3 | 22.15 | 0.0117 | Colipase, pancreatic |  |
| Tjp1 | 21872 | 231717 | 7 C | 2.41 | 0.048 | Tight junction protein 1 |  |
| Cldn1 | 12737 | 15195 | 16 B2 | 1.57 | 0.006 | Claudin 1 |  |
| Ocln | 18260 | 56215 | 13 D1 | 1.56 | 0.034 | Occludin |  |
| Fabp2 | 14079 | 4435 | 3 G1 | 1.59 | 0.023 | Atty acid binding protein 2, intestinal |  |
| Hba-a2 | 110257 | 819 | 11 A4 | 1.55 | 0.001 | Hemoglobin alpha, adult chain 2 |  |
| Nfkbia | 18035 | 3239 | 12 C1 | 1.8 | 0.004 | Nuclear factor of kappa light polypeptide gene enhancer in B cells inhibitor, alpha |  |
| Cldn5 | 12741 | 1416 | 16 A3 | 1.52 | 0.006 | Claudin 5 |  |
| Hbb-bs | 100503605 | 1406 | 7 E3 | 1.71 | 0.001 | Hemoglobin, beta adult s chain |  |
| Dgat2 | 67800 | 29051 | 7 E1 | 1.58 | 0.01 | Diacylglycerol O-acyltransferase 2 |  |
| Hmgcs2 | 15360 | 30308 | 3 F2.2 | 1.87 | 0.024 | 3-hydroxy-3-methylglutaryl-Coenzyme A synthase 2 |  |
| Sgk2 | 27219 | 26811 | 2 H2 | 1.77 | 0.001 | Serum/glucocorticoid regulated kinase 2 |  |
| Bcl3 | 12051 | 14305 | 7 A3 | 1.84 | 0.029 | B cell leukemia/lymphoma 3 |  |
| Igfbp3 | 16009 | 9413 | 11 A1 | 1.54 | 0.008 | Insulin-like growth factor binding protein 3 |  |
| Ccl21d | 100862177 | 8557 | 4 A5 | 1.65 | 0.001 | Chemokine (C-C motif) ligand 21D |  |
| Hba-a1 | 15122 | 815 | 11 A4 | 1.68 | 0.001 | Hemoglobin alpha, adult chain 1 |  |
| H2-T22 | 15039 | 5615 | 17 B1 | 0.58 | 0.044 | Histocompatibility, T region locus 22 |  |
| Cdk1 | 12534 | 17767 | 10 B5.3 | 0.67 | 0.001 | Cyclin-dependent kinase 1 |  |
| Hmgcs1 | 208715 | 17910 | 13 D2.3 | 0.63 | 0.001 | 3-hydroxy-3-methylglutaryl-Coenzyme A synthase 1 |  |
| Cdc25b | 12531 | 12076 | 2 F1 | 0.61 | 0.001 | Cell division cycle 25B |  |
| Atp1a3 | 232975 | 27911 | 7 A3 | 0.67 | 0.017 | ATPase, Na+/K+ transporting, alpha 3 polypeptide |  |
| H2-Q6 | 110557 | 5206 | 17 B1 | 0.66 | 0.015 | Histocompatibility, Q region locus 6 |  |
| Plk4 | 20873 | 16933 | 3 B | 0.64 | 0.01 | Polo like kinase 4 |  |
| Il1b | 16176 | 6570 | 2 F1 | 0.63 | 0.005 | Interleukin 1 beta |  |
| Ccnb2 | 12442 | 13866 | 9 D | 0.59 | 0.001 | Cyclin B2 |  |
| Ccnb1 | 268697 | 7748 | 13 D1 | 0.54 | 0.001 | Cyclin B1 |  |
| Casp1 | 12362 | 8765 | 9 A1 | 0.53 | 0.026 | Caspase 1 |  |
| Nlrp3 | 216799 | 25387 | 11 B1.3 | 0.51 | 0.009 | NLR pyrin domain containing 3 |  |

**Table S3: Six machine learning models prediction results.**

| Gene Symbol | LASSO | RandomForest | XGBoost | Boruta | SVM | Bayes | Sum |
| --- | --- | --- | --- | --- | --- | --- | --- |
| NRF2 | TRUE | TRUE | TRUE | TRUE | TRUE | TRUE | 6 |
| RORC | TRUE | TRUE | TRUE | TRUE | TRUE | TRUE | 6 |
| CAMK2A | TRUE | TRUE | TRUE | TRUE | TRUE | TRUE | 6 |
| SLC6A11 | TRUE | TRUE | FALSE | TRUE | TRUE | TRUE | 5 |
| FABP5 | TRUE | TRUE | TRUE | FALSE | TRUE | FALSE | 4 |
| RXRA | TRUE | FALSE | TRUE | FALSE | TRUE | TRUE | 4 |
| CDC25B | TRUE | TRUE | TRUE | FALSE | TRUE | FALSE | 4 |
| GNB1 | TRUE | TRUE | FALSE | FALSE | TRUE | TRUE | 4 |
| ARG2 | TRUE | TRUE | FALSE | TRUE | FALSE | FALSE | 3 |
| MMP1 | TRUE | TRUE | FALSE | FALSE | FALSE | TRUE | 3 |
| PTGS1 | TRUE | FALSE | FALSE | FALSE | TRUE | TRUE | 3 |
| MAOA | TRUE | TRUE | FALSE | FALSE | TRUE | FALSE | 3 |
| MAOB | TRUE | TRUE | FALSE | FALSE | TRUE | FALSE | 3 |
| NOS1 | TRUE | TRUE | FALSE | TRUE | FALSE | FALSE | 3 |
| HDAC1 | TRUE | FALSE | FALSE | TRUE | FALSE | TRUE | 3 |
| TLR2 | TRUE | TRUE | FALSE | FALSE | TRUE | FALSE | 3 |
| VEGFA | TRUE | FALSE | FALSE | FALSE | TRUE | TRUE | 3 |
| THRA | TRUE | FALSE | FALSE | FALSE | TRUE | TRUE | 3 |
| NOS2 | TRUE | TRUE | FALSE | FALSE | FALSE | FALSE | 2 |
| SPHK1 | TRUE | TRUE | FALSE | FALSE | FALSE | FALSE | 2 |
| GABRQ | TRUE | FALSE | FALSE | FALSE | TRUE | FALSE | 2 |
| TREM2 | TRUE | FALSE | FALSE | FALSE | FALSE | TRUE | 2 |
| GRIK1 | TRUE | FALSE | FALSE | FALSE | TRUE | FALSE | 2 |
| GRIA1 | TRUE | FALSE | FALSE | FALSE | TRUE | FALSE | 2 |
| GRIA3 | TRUE | FALSE | FALSE | FALSE | FALSE | TRUE | 2 |
| RELA | TRUE | FALSE | FALSE | FALSE | TRUE | FALSE | 2 |
| NFKB1 | TRUE | TRUE | FALSE | FALSE | FALSE | FALSE | 2 |
| EP300 | TRUE | FALSE | FALSE | FALSE | TRUE | FALSE | 2 |
| PPARA | TRUE | TRUE | FALSE | FALSE | FALSE | FALSE | 2 |
| MAPT | TRUE | TRUE | FALSE | FALSE | FALSE | FALSE | 2 |
| NOD2 | TRUE | TRUE | FALSE | FALSE | FALSE | FALSE | 2 |
| DUSP3 | TRUE | FALSE | FALSE | FALSE | FALSE | TRUE | 2 |
| NOS3 | TRUE | FALSE | FALSE | FALSE | FALSE | TRUE | 2 |
| IL17RA | TRUE | TRUE | FALSE | FALSE | FALSE | FALSE | 2 |
| CPT2 | TRUE | FALSE | FALSE | FALSE | TRUE | FALSE | 2 |
| JUN | TRUE | FALSE | FALSE | FALSE | FALSE | TRUE | 2 |
| FOS | TRUE | FALSE | FALSE | FALSE | FALSE | TRUE | 2 |
| ESR2 | TRUE | FALSE | FALSE | FALSE | FALSE | TRUE | 2 |
| ALOX12 | FALSE | FALSE | TRUE | FALSE | FALSE | FALSE | 1 |
| PRKCA | FALSE | FALSE | FALSE | FALSE | FALSE | TRUE | 1 |
| GABBR2 | TRUE | FALSE | FALSE | FALSE | FALSE | FALSE | 1 |
| GABBR1 | TRUE | FALSE | FALSE | FALSE | FALSE | FALSE | 1 |
| PTGS2 | TRUE | FALSE | FALSE | FALSE | FALSE | FALSE | 1 |
| IL2 | TRUE | FALSE | FALSE | FALSE | FALSE | FALSE | 1 |
| HRAS | TRUE | FALSE | FALSE | FALSE | FALSE | FALSE | 1 |
| HCAR2 | TRUE | FALSE | FALSE | FALSE | FALSE | FALSE | 1 |
| NDUFA1 | TRUE | FALSE | FALSE | FALSE | FALSE | FALSE | 1 |
| FABP4 | TRUE | FALSE | FALSE | FALSE | FALSE | FALSE | 1 |
| PTGES | FALSE | FALSE | FALSE | FALSE | FALSE | TRUE | 1 |
| ACT1 | FALSE | FALSE | FALSE | FALSE | FALSE | FALSE | 0 |
| KYNU | FALSE | FALSE | FALSE | FALSE | FALSE | FALSE | 0 |
| GABRR1 | FALSE | FALSE | FALSE | FALSE | FALSE | FALSE | 0 |

**Table S4: The docking energies of ACT and FA with Key target of microglia-mediated**

**neuroinflammation.**

| **Ingredient** | **Abbreviation** | **Molecular weight** | **Key target of microglia-mediated neuroinflammation (kcal/mol)** | | | | | | |
| --- | --- | --- | --- | --- | --- | --- | --- | --- | --- |
|  |  |  | RORγt | IL-17RA | Act1 | TRAF6 | Nrf2 | TREM2 | DAP12 |
| Acetoside | ACT | 624.59 | -7.8 | -7.6 | -6.7 | -7.2 | -9.3 | -7.8 | -6.3 |
| Ferulic acid | FA | 194.18 | -7.2 | -5.7 | -5.3 | -5.3 | -6.6 | -5.7 | -5.9 |

**Table S5: Gene primer sequence information.**

| **Genes name** | **Forward Primer Sequence** | **Reverse Primer Sequence** |
| --- | --- | --- |
| **Nrf2** | 5’-AAAATCATTAACCTCCCTGTTGAT-3’ | 5’-CGGCGACTTTATTCTTACCTCTC-3’ |
| **TREM2** | 5’-CTGGAACCGTCACCATCACTC-3’ | 5’-CGAAACTCGATGACTCCTCGG-3’ |
| **DAP12** | 5’-AAGATGCGACTGTTCTTCCGT-3’ | 5’-CCAGGGCAATCAGCAGAGTC-3’ |
| **Arg-1** | 5’-CTCCAAGCCAAAGTCCTTAGAG-3’ | 5’-AGGAGCTGTCATTAGGGACATC-3’ |
| **RORyt** | 5’-GGAAACCAGGCATCCTGAAC-3' | 5'-GCACTGCAGAAACTGGGAATG-3' |
| **IL17ra** | 5'-AGTTCCCAAGCCAGTTGCAG-3' | 5’-AGCACGATGACAGATCCCAC-3’ |
| **ACT1** | 5’-CTTTCTACGTTTCCATTCAAGCTG-3' | 5'-GGAGTTTGACCCACCTCTGG-3’ |
| **Traf6** | 5'-GGAGTTTGACCCACCTCTGG-3’ | 5’-CTTGTGCCCTGCATCCCTTA-3’ |
| **Iba-1** | 5’-CGGGATCCGAGCTATGAGCCAGAGCAAG-3' | 5'-GGAATTCCCCACCGTGTTATATCCACC-3' |
| **iNOS** | 5’-ACAACAGGAACCTACCAGCTCA-3’ | 5’-GATGTTGTAGCGCTGTGTGTCA-3’ |
| **GAPDH** | 5’-CGTCCCGTAGACAAAATGGT-3’ | 5’--TTGATGGCAACAATCTCCAC-3’ |
| **Si-Nrf2** | CGAGAAGUGUUUGACUUUATT | UAAAGUCAAACACUUCUCGTT |
